# The puzzle of profitless pre-cues

**DOI:** 10.3758/s13423-025-02841-z

**Published:** 2026-03-18

**Authors:** Julie M. Bugg, Christopher O. Nuño, Changrun Huang, Tobias Egner

**Affiliations:** 1 Washington University in St. Louis, St. Louis, MO USA; 2 Duke University, Durham, NC USA

**Keywords:** Pre-cues, Conflict tasks, Cognitive control, Proactive control

## Abstract

Despite the intuition that attention can be willfully heightened on demand in response to warnings that alert people to impending distraction (e.g., “Pay attention!”), the empirical evidence for this notion from studies employing congruency pre-cues is surprisingly weak, posing a challenge to some theoretical accounts. Here, we examine this puzzle of profitless pre-cues via the first systematic review of the literature on pre-cues in conflict tasks (e.g., the Stroop task)—a classic instance of proactive cognitive control. We first outline important conceptual and methodological considerations to delineate the process of greatest theoretical and practical importance, namely, the volitional attenuation of distraction when the precise target and distractor features are not known in advance. This is followed by a comprehensive literature review, revealing limited evidence for the effective use of pre-cues to attenuate distraction in conflict tasks (and we note a similar status in adjacent fields, like task switching and visual search). To elucidate this puzzle, we synthesize key findings alongside design parameters employed across studies to develop a novel theoretical framework, called TEPID. TEPID proposes a two-phase model that highlights the interactivity of a small set of task-related factors (preparation time, cue type, response modality, and task type) and person-related factors (motivation and ability) which we believe to be crucial for determining whether pre-cues are exploited to proactively modulate control. From this synthesis, several recommendations and predictions are derived to position the field for future investigations of proactive control using pre-cueing manipulations, as well as their translation to real-life applications.

Cognitive control is the ability to guide thoughts and actions in alignment with one’s goals. A central component of cognitive control is the adaptation of attention in response to varying external demands. To achieve goals, people need to heighten attentional focus in the presence of distraction, directing more attention toward goal-relevant information and/or filtering out irrelevant information. The omnipresent imperative “Pay attention!” implies that people believe others can indeed be more focused when instructed to do so. Examples are abundant. In the classroom, teachers implore students to pay more attention. On the highway, both road signs and dashboard warnings instruct drivers to not get distracted. In the workplace, managers direct employees to focus harder as a deadline looms, and coaches direct professional athletes to focus on their game and not be distracted by an opponent’s antics. Yet, despite the strong intuition that attention can be willfully heightened on demand in situations like these, the empirical evidence for this notion is surprisingly weak. The purpose of this paper is to systematically review that evidence, focusing primarily on research that has utilized pre-cues—verbal or symbolic “warnings” or “hints” that provide attention-directing information about an upcoming stimulus—in conflict tasks (e.g., the Stroop task) to examine the proactive control of attention in the face of expected distraction. In so doing, we aim to develop an understanding of factors that may explain the weak evidence to date for pre-cue benefits and position the field to provide further tests of the benefits of pre-cues under conditions that are optimized to reveal them, according to our synthesis of the literature.

Our review is organized as follows. First, we describe what we mean by the puzzle of profitless pre-cues and the significance of this puzzle, practically and theoretically. Then, we provide an overview of key paradigms, outcomes, and possible mechanisms within the pre-cueing literature. Subsequently, we present a comprehensive review of the extant pre-cueing literature, which we then use to develop a novel theoretical framework that highlights critical factors proposed to dictate pre-cueing effects, a model of their interactivity, and testable predictions that may serve as a roadmap for future research. We then draw connections to related literatures where researchers have similarly questioned how effectively people can willfully enhance attention based on pre-cues (or other hints provided in advance of a stimulus) before discussing practical and theoretical implications.

## What is the puzzle of profitless pre-cues and its significance?

Pre-cues have been used in multiple literatures, with Posner’s studies of spatial attention in the 1980s being perhaps the most well-known example (Posner, 1980; Posner et al., 1980). In these studies, a target stimulus could appear on the left or right side of a screen, and participants pressed a response key as soon as they detected the stimulus, regardless of its location. If, prior to stimulus presentation, a pre-cue (an arrow pointing left or right) validly indicated the location at which the target would appear, response times were speeded relative to uncued (neutral) and invalidly cued conditions. Thus, participants seem to successfully translate spatial information provided by a pre-cue into a (covert) shift of attention to the cued location. This type of pre-cue benefit on spatial orienting has been observed in dozens of studies (for a review, see Chica et al., 2014), and a similar picture holds for the pre-cueing of attention to simple nonspatial stimulus features, as people readily use prior information about stimulus color or shape to find targets among distractors (Egeth et al., 1984; Kaptein et al., 1995).

However, pre-cue benefits are much more elusive when moving from cues that provide information about single, concrete stimulus features (like target location or color) to cues that provide more abstract, relational information about stimulus features (i.e., whether the relevant and irrelevant dimensions will conflict or match), which is typically the case in studies of cognitive control. Pre-cue benefits are especially elusive in conditions that most closely map onto the examples provided at the outset: when distraction is expected but it is not certain what the distraction will entail. Here, a pre-cue benefit depends on people biasing attention in a nonspecific fashion to minimize the detrimental effects of anticipated distraction. The on-demand heightening of attention in these conditions is the topic of the current review.

Specifically, we focus on *congruency pre-cues* in conflict tasks, namely the Stroop, flanker, and Simon tasks. While these tasks vary in several ways, what they have in common is that participants are instructed to attend to a target (the relevant dimension; e.g., color in color-word Stroop; a central target such as an arrow in flanker; the identity of a stimulus in Simon) while ignoring distractor(s) (the irrelevant dimension; e.g., a word in color-word Stroop; flanking stimuli such as arrows in flanker; the location of a stimulus in Simon), and they incur a processing cost when the two dimensions conflict (incongruent trials) compared with when they align (congruent trials), resulting in a congruency effect (slowing on incongruent relative to congruent trials).^1^ In all these tasks, researchers have examined whether being warned via a pre-cue that the two dimensions will conflict (i.e., in lay terms, being told to pay attention because distraction is imminent) leads to better performance than when one is not warned (i.e., an uncued comparison condition).

A congruency pre-cue validly signals the congruency status of an upcoming trial (or as we will detail later, an upcoming list of trials).^2^ Congruency pre-cues sometimes comprise words that directly communicate to participants what the next trial (or list) will be (e.g., CONFLICTING indicates an incongruent trial, MATCHING indicates a congruent trial), which we will refer to as *semantic* pre-cues. Pre-cues can also be *symbolic*, for example, participants might be told that a certain shape or color signals the congruency of the next trial (e.g., a circle indicates an incongruent trial, a diamond indicates a congruent trial). Importantly, regardless of cue type, the information provided by congruency pre-cues does not refer to a specific, concrete stimulus feature (like the color “red” or word “BLUE” in the color-word Stroop task) but to the relationship (e.g., conflicting or matching) between two stimulus dimensions (e.g., color and word information in the Stroop task). In theory, this type of pre-cue nevertheless allows people to anticipate and prepare for upcoming distraction on incongruent trials, thereby presumably enhancing performance compared with when this information is not provided (uncued condition). For example, a participant may try to suppress attention to the word and/or increase their focus on color processing in response to a pre-cue signaling an incongruent trial in the Stroop task.

The puzzle of profitless pre-cues refers to the question of why there are often no pre-cue benefits observed on incongruent trials and mostly incongruent lists, as we will detail in the “Review of Literature” section. In other words, it is puzzling that response times in the cued and uncued condition are often equivalent even though the pre-cues are 100% valid. This pattern stands in stark contrast to the pre-cue benefits observed in paradigms such as those employed by Posner, and to the intuition that people can heighten attentional focus on demand and therefore should benefit from the provision of a congruency pre-cue.

The lack of pre-cue benefits is even more puzzling given that there are highly reliable behavioral signatures of adjustments in cognitive control observed in the very same tasks used in pre-cueing experiments, adjustments that are based on experience rather than pre-cueing. For example, people show reduced congruency effects on trials following an incongruent trial (i.e., the congruency sequence effect, reviewed in Egner, 2017), in lists (blocks) of trials where incongruent trials are frequent compared with when they are rare (i.e., the list-wide proportion congruent effect, reviewed in Bugg & Crump, 2012; Egner, 2023), and for items (stimulus features) that tend to be incongruent rather than congruent (i.e., the item-specific proportion congruence effect, reviewed in Bugg, 2012, 2017). Thus, the central question at the heart of the puzzle is: Why are congruency pre-cues (or pre-cues for short, hereafter) signaling that distraction is imminent, typically profitless?

Solving this puzzle is not only important from an applied perspective in contexts like those described above (i.e., teaching, driving, and workplace settings), it is also important from a theoretical perspective. The notion that people can willfully adjust attention (or action) as needed, generally considered a conscious (i.e., intentional, voluntary) process, is a signature of many early theories of cognitive control (e.g., Ach, 1910; Norman & Shallice, 1986; Posner & Snyder, 1975; Shiffrin & Schneider, 1977). For example, Norman and Shallice (1986) posited the existence of a supervisory attention system that monitors for conflict and willfully biases attention in a goal-directed fashion. On such views, one would expect that people can harness pre-cues to their benefit, volitionally adjusting attention to minimize distraction. The same expectation emerges when considering a current and prominent view of cognitive control, the Dual Mechanisms of Control (DMC) account (Braver, 2012; Braver et al., 2007). According to the DMC account, people can reactively control attention to cope with distraction (i.e., after a stimulus grabs their attention) or proactively control attention to prevent or minimize distraction (i.e., before a stimulus grabs their attention). Most relevant to the present puzzle, the DMC account assumes that people employ proactive control when they can reliably anticipate the occurrence of future distractions. Most pre-cueing paradigms use 100% valid pre-cues and thus enable participants to do exactly this, yet pre-cue benefits rarely emerge. It remains uncertain whether people are capable of, but choose not to, willfully utilize pre-cues to prepare attention prior to a stimulus, or alternatively, are not capable of doing so. We suspect it is a mixture of these explanations and others, as we will elaborate. Either way, the puzzle questions the ubiquity of proactive control.

## Pre-cueing paradigms and primary outcomes of interest

**Fig. 1:**
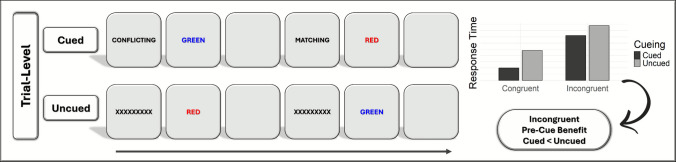
General trial structure for trial-level pre-cueing paradigm. Although pre-cue type varies across experiments, as detailed in Tables 1 and 2, the semantic pre-cues depicted here are from Bugg and Smallwood (2016). The blank screen corresponds to the preparatory interval, which also varies across studies (see Tables 1 and 2). Although only two trials are illustrated for each condition (cued and uncued), participants see the words RED, BLUE, GREEN, and YELLOW paired with the colors red, blue, green, and yellow in a four-choice color-word Stroop task. The plot on the right displays the hypothetical trial-level pre-cue benefits, with response time on the *y*-axis and trial type (congruent vs. incongruent) on the *x*-axis. If participants use pre-cues in a four-choice task to prepare and implement a distractor-attenuating strategy on incongruent trials, then a pre-cue benefit is expected, which is illustrated by the speeding of response times in the cued (i.e., informative) compared with the uncued (i.e., uninformative) condition. (Color figure online)

**Fig. 2:**
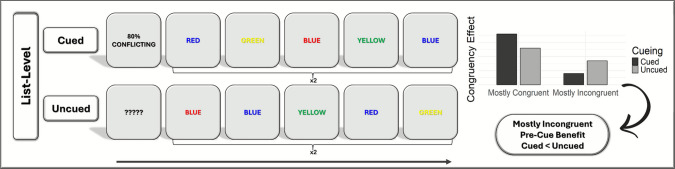
General trial structure for list-level pre-cueing paradigm. Only semantic pre-cues have been used in list-level studies, as detailed in Table 3, and the pre-cues shown here are from Bugg et al. (2015). Only one list is illustrated for each condition (cued and uncued), in a four-choice color-word Stroop task. Given that lists generally consist of 10 trials, we used “x2” to indicate that the list typically extends beyond the five trials shown. Across lists, participants see the words RED, BLUE, GREEN, and YELLOW paired with the colors red, blue, green, and yellow. Typically, a blank screen is presented between stimuli, though it is not illustrated here. The plot on the right displays hypothetical list-level pre-cueing effects, with the congruency effect (i.e., Stroop effect) on the *y*-axis and list type (mostly congruent vs. mostly incongruent) on the *x*-axis. If participants use pre-cues in a four-choice task to prepare and implement a distractor-attenuating strategy for mostly incongruent lists, then a pre-cue benefit is expected, which is illustrated by the reduction in the congruency effect for cued (i.e., informative) compared with uncued (i.e., uninformative) lists. (Color figure online)

There are two primary pre-cueing paradigms that have been used in this literature: the *trial-level pre-cueing* paradigm and the *list-level pre-cueing* paradigm (see Figs. 1 and 2, respectively, for illustrations of these paradigms in the context of the color-word Stroop task). In the trial-level paradigm, the pre-cue accurately informs participants about the congruency of the trial that immediately follows the pre-cue, indicating whether the next trial will be incongruent (e.g., the pre-cue CONFLICTING) or congruent (e.g., the pre-cue MATCHING). In the list-level paradigm, the pre-cue accurately informs participants about the proportion congruency of the list that immediately follows the pre-cue, with lists typically comprising 10 trials. Here, pre-cues signal whether the list of trials will be mostly incongruent (e.g., the pre-cue 80% CONFLICTING) or mostly congruent (e.g., the pre-cue 80% MATCHING). In both paradigms, in addition to the cued condition, there is an uncued condition in which participants are not informed about the congruency of the next trial or proportion congruency of the list. Typically, in the uncued condition participants are presented with an uninformative pre-cue, such as a string of symbols prior to each trial (e.g., XXXXXXXXX) or at the start of each list (e.g., ?????) in place of the congruency pre-cue. Critically, these symbols are not predictive; rather, they are equally likely to be followed by an incongruent/congruent trial or a mostly incongruent/mostly congruent list. The uncued condition serves as a baseline against which performance in the cued condition can be evaluated because these two conditions are the same except for the cues. Generally, the informative pre-cues are intermixed in one block and compared with uninformative pre-cues in another block, or all pre-cues are randomly intermixed.

While the trial-level and list-level pre-cueing paradigms are used to address the same overarching question concerning the on-demand adjustment of control based on advance information, there are two differences we wish to highlight. The first difference regards what information is communicated by the pre-cue. In the trial-level case the pre-cue signals congruency whereas in the list-level case the pre-cue signals proportion congruency. Thus, trial-level pre-cues are deterministic, meaning that it is certain what the congruency of the next trial will be. In contrast, list-level pre-cues are probabilistic, meaning that although the pre-cues are 100% valid, it is uncertain what the congruency of a given trial will be, but it is more likely to be in accordance with the pre-cue (e.g., incongruent for mostly incongruent pre-cue).

The second difference is that the two paradigms target adjustments in control that vary in temporal scope (transient vs. sustained). The trial-level paradigm enables researchers to index people’s ability to heighten control in a transient manner, meaning on a single trial. To gain a pre-cue benefit, one needs to adjust control on each trial but maintain that level of control only for a brief period (one trial). In contrast, the list-level paradigm enables researchers to index people’s ability to heighten control in a more temporally extended, sustained way. To gain a pre-cue benefit at the list-level, one needs to adjust control only at the start of each list but then sustain that level of control across multiple trials. Given this difference in temporal scope, participants must exercise a higher degree of preparatory flexibility (in selecting and preparing a relevant strategy in response to pre-cues, which presumably demands updating and switching processes) in the trial-level paradigm than the list-level paradigm.

The primary outcome of interest in both pre-cueing paradigms is the *pre-cue benefit*, which is indexed by comparing performance in the cued condition to the uncued condition. However, the two paradigms draw different comparisons. In the trial-level paradigm, researchers compare response times between the cued and uncued condition for each trial type (incongruent and congruent trials) separately; in the list-level paradigm, researchers compare the congruency effect (i.e., difference between incongruent and congruent trials; also called a compatibility effect, or a Stroop, flanker, or Simon effect) between the cued and uncued condition. The rationale is as follows: In the trial-level paradigm, when 100% valid pre-cues are used, incongruent trials selectively follow pre-cues signaling an upcoming incongruent trial (and congruent trials selectively follow congruent pre-cues). Accordingly, it is not possible to derive a congruency effect for each informative pre-cue (CONFLICTING and MATCHING), and thus one cannot compare congruency effects between the cued and uncued condition. In the list-level paradigm, the rationale for comparing the congruency effect between cued and uncued lists is that, unlike in the trial-level paradigm, trials of each congruency level (i.e., trial type) follow each of the possible pre-cues (e.g., the 80% CONFLICTING, 80% MATCHING, and ????? pre-cues are both followed by congruent and incongruent trials, although with different frequencies), such that either trial type can be affected by adjustments made in response to a given pre-cue.

In the trial-level paradigm, of most importance theoretically and practically is the question of whether a pre-cue benefit is observed for incongruent trials, which is indicated by faster response times in the cued condition compared with the uncued condition. Pre-cue benefits on incongruent trials uniquely allow researchers to make the inference that participants utilized advance information proactively to minimize effects of the distractor, because the task-relevant and task-irrelevant stimulus features conflict on these trials. Pre-cue benefits on congruent trials (calculated by comparing the cued to uncued condition for congruent trials) are of less interest, because they do not permit this inference since the two features match. Additionally, a pre-cue benefit on congruent trials may indicate that participants attended *more* to the distractor in the cued condition (i.e., used a distractor-enhancing strategy as we will detail in the next section “Mechanisms Underlying Pre-Cue Benefits”).

In the list-level paradigm, of most importance theoretically and practically is whether a pre-cue benefit is observed for mostly incongruent lists, and in this case, it is indicated by a reduction in the mean (i.e., list-level) congruency effect in cued mostly incongruent lists compared with uncued mostly incongruent lists. This benefit, like the pre-cue benefit for incongruent trials in the trial-level paradigm, allows researchers to infer that participants utilized the pre-cues to minimize the effects of the anticipated distraction. For example, if attention is heightened and sustained in response to a pre-cue signaling a mostly incongruent list, this should be reflected in a reduced congruency effect in the cued list (i.e., due to speeding on incongruent and/or slowing on congruent trials) compared with the uncued list. However, as we will detail in our literature review, there is thus far no evidence of a pre-cue benefit at the list level.

Though the effects of pre-cues in mostly congruent lists are of less interest in the present review for the same reasons that effects on congruent trials in the trial-level paradigm are of less interest, we briefly note that such effects also are evaluated by comparing congruency effects between cued and uncued lists. When a cue signals a mostly congruent list, the expectation is that participants will relax, rather than heighten control, because the list comprises more congruent (i.e., easier) trials. When control is relaxed, more attention is allocated to the distractor (irrelevant dimension), such that the rare incongruent trials slow performance even more than is typical. Accordingly, pre-cue use in mostly congruent lists should result in a larger congruency effect for cued than uncued lists. That is, there should be a *pre-cue cost* for the mostly congruent condition. This pattern has been reliably observed in the literature (Bugg & Diede, 2018; Bugg et al., 2015; Nuño et al., 2025; Suh et al., 2022).

In addition to comparing mean congruency effects between lists, researchers have also analyzed pre-cue benefits selectively on the list’s first trial by comparing the congruency effect between cued and uncued lists. The rationale is that performance on the first trial of a list (i.e., first trial following the pre-cue) is the purest indicator of pre-cue use, independent of any adaptations of control that occur based on experience within the list (i.e., exposure to incongruent or congruent trials, which by way of congruency sequence effects or list-wide proportion congruency effects, may increase or decrease control). A pre-cue benefit in first-trial analyses is indicative of a transient (rather than sustained) adjustment, as in the trial-level paradigm, though in the list-level paradigm, the congruency of the first trial is only probabilistically determined by the pre-cue.

## Mechanisms underlying pre-cue benefits

A central question regards the mechanisms purported to underlie pre-cue benefits in conflict tasks. Although above we noted that observing a pre-cue benefit on incongruent trials or in mostly incongruent lists uniquely affords the inference that participants utilized the pre-cues to minimize distraction, a key question is what exact mechanism(s) lead to pre-cue benefits. In other words, what does it mean to say that participants utilized the pre-cue to enhance performance? The answer to this question is nuanced and brings us to an important point: whether pre-cue benefits are a valid signature of the on-demand heightening of control depends on the task design. Specifically, it matters whether congruency pre-cues are delivered in a two-choice task design or in a task with more than two choices—a distinction that is crucial, both theoretically and practically.

First, as noted in a previous section (“What is the Puzzle of Profitless Pre-Cues and its Significance?”), the attentional mechanism underlying the successful use of pre-cues must be *nonspecific* (abstract) in the sense that the pre-cue does not supply information about a specific, concrete stimulus or response feature. This type of nonspecific attentional biasing is thought to operate at the level of stimulus dimensions and is sometimes referred to as “dimensional gating” (Wühr & Kunde, 2008; see also Botvinick et al., 2001; Cohen et al., 1990). The idea is that people use pre-cues signaling an incongruent trial or mostly incongruent list to prepare an identity-blind or content-independent filter that operates on stimulus dimensions rather than specific features. This can involve down-weighting task-irrelevant dimensions (word in color-word Stroop tasks, flanking stimuli in flanker tasks, location in Simon tasks) and amplifying task-relevant dimensions (color in color-word Stroop tasks, central position in flanker tasks, relevant stimulus dimension in Simon tasks). To be clear, nonspecific means that the bias is not directed away from a specific feature (e.g.,, ignoring the word BLUE or focusing harder on the color red in the Stroop task). Such adjustments are not afforded by congruency pre-cues since these pre-cues signal only congruency and not the specific stimulus features that will appear on the next trial.^3^

**Fig. 3:**
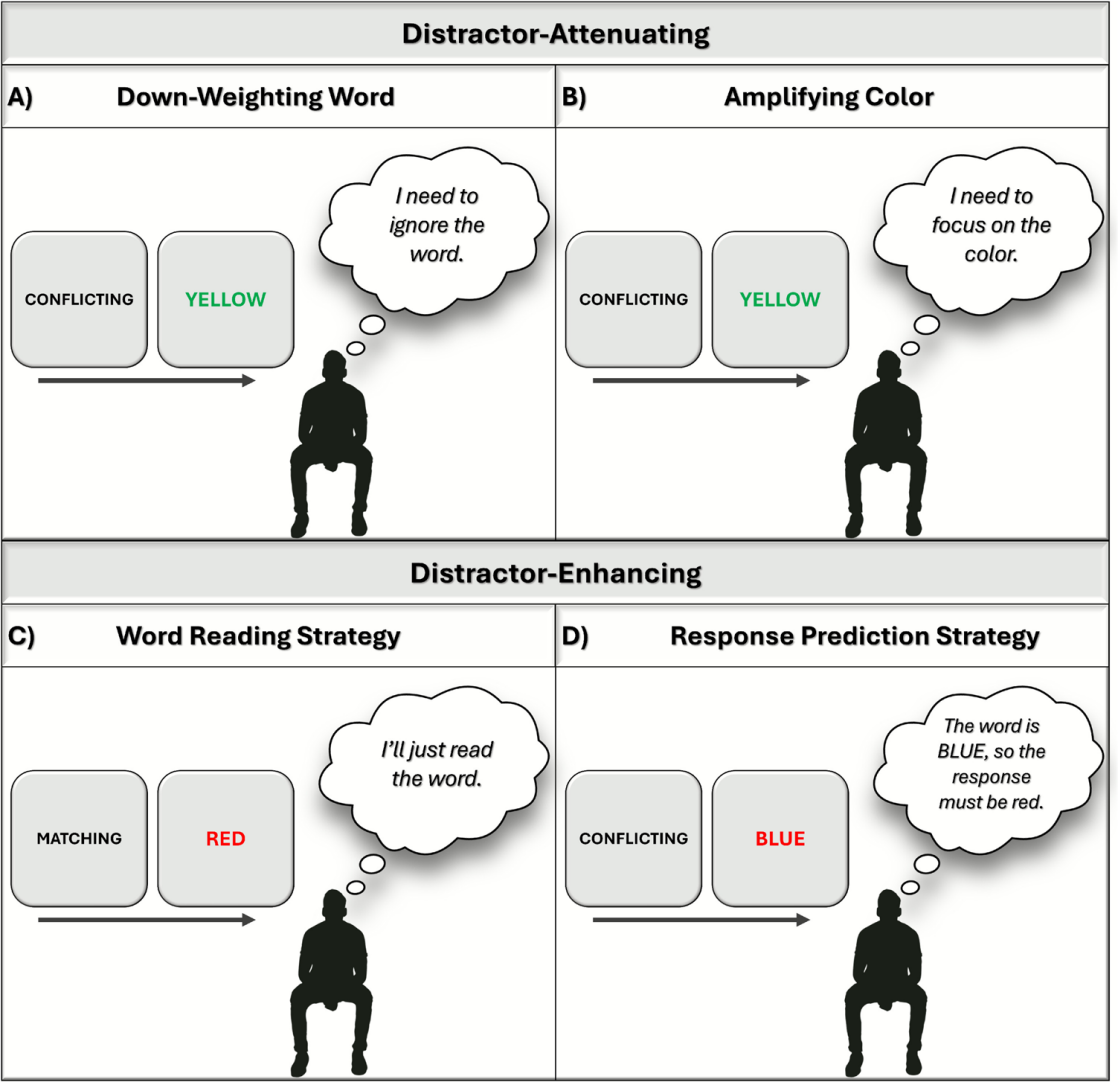
Visualization of the attentional mechanisms underlying congruency pre-cue use using the Stroop task as an example. Panels **A** and **B** illustrate the distractor-attenuating strategy, which may entail down-weighting of the word dimension and/or amplifying attention to the color dimension. While a distractor-attenuating strategy can theoretically be applied to two-choice and four-choice tasks, the examples in Panels **A** and **B** assume a four-choice task where the strategy is necessary to gain a pre-cue benefit on incongruent trials (or in mostly incongruent lists) because the identity of the distractor (i.e., “YELLOW”) is not predictive of the correct response (i.e., green). A nonspecific (abstract) down-weighting of the distractor (i.e., the word dimension) is depicted in Panel **A**, whereas a nonspecific (abstract) amplification of the target (i.e., the color) is depicted in Panel **B**. Panels **C** and **D** illustrate the distractor-enhancing strategies. The word-reading strategy is depicted in Panel **C**, where a participant can use a pre-cue signaling a congruent trial to read the word in the Stroop task. The response prediction strategy is depicted in Panel **D**, where a participant can use the pre-cue signaling an incongruent trial to produce the response opposite to the word. Importantly, this strategy is only possible in a two-choice task (e.g., comprising colors blue/red and words BLUE/RED). These two strategies are considered distractor-enhancing because the participant attends to the irrelevant dimension (distractor) to produce the response. Note that although we used the Stroop task to illustrate the different strategies in this figure, as described in the text, the distractor-attenuating and distractor-enhancing strategies can be applied to other conflict tasks, such as the flanker and Simon task (though word reading is an example of a distractor-enhancing strategy specific to Stroop; in flanker or Simon, this would instead entail directing attention to the distractor and directly responding based on its identity). Additionally, as described in the text and Footnote 5, we assume that similar mechanisms illustrated in the figure are applicable to the list-level paradigm, when cues signal mostly congruent or mostly incongruent lists, rather than congruent or incongruent trials. (Colof figure online)

Second, it is important to distinguish this nonspecific attentional bias, which we will refer to hereafter as a *distractor-attenuating*^4^ strategy, from alternative *distractor-enhancing* strategies, which entail intentionally directing attention to the irrelevant dimension (i.e., switching attention; Logan & Zbrodoff, 1982; Wühr & Kunde, 2008). These strategies are illustrated in the context of the color-word Stroop task in Fig. 3. The distinction between distractor-attenuating and distractor-enhancing strategies is important because participants can use a distractor-enhancing strategy to gain a pre-cue benefit in conditions where the pre-cue signals a congruent trial regardless of task design (two-choice or more than two-choice),^5^ and when a pre-cue signals an incongruent trial uniquely in two-choice task designs. One distractor-enhancing strategy is reading the word, and this strategy can in principle be applied whenever one expects the upcoming trial to be congruent, and in this case, the response is based directly on the identity of the distractor (see Fig. 3C). Less obvious but of importance, a distractor-enhancing strategy can also be applied when an incongruent trial is expected and the stimulus and response set is small—specifically in a two-choice task where there are only two response options and two task-relevant and task-irrelevant stimulus feature values. In this case, the identity of the distractor (e.g., the word in color-word Stroop) can be used to directly predict the identity of the relevant dimension (the color), and thus, the correct response. We refer to this as a *response prediction strategy*.

For example, a two-choice Stroop task might entail the words BLUE and RED and the colors blue and red. Assume that a pre-cue signaling an incongruent trial is presented followed by BLUE in red ink, as in Fig. 3D. Since there are only two possible responses, and the pre-cue indicates that the color (correct response) will not be the same as the word (i.e., it will be the opposite response), a participant can attend to the irrelevant dimension (the word BLUE) and use its identity to determine that the relevant dimension (color) is red. That is, they can predict the opposite response. Similarly, in a two-choice flanker task or Simon task, participants can use the flankers or stimulus location to predict the correct (i.e., opposite) response when they expect an incongruent trial. In the uncued condition, pre-cues are not predictive of trial type; therefore, this distractor-enhancing strategy does not allow participants to accurately predict the identity of the relevant dimension (i.e., the word BLUE is just as likely to be associated with the color blue as it is the color red following an uninformative pre-cue). Accordingly, pre-cue benefits on incongruent trials in two-choice task designs may be wholly driven by the response prediction strategy, which could be attractive to employ because it allows the participant to use the more automatically (less effortfully) processed stimulus feature to drive their choices. However, because response prediction is a distractor-enhancing strategy rather than a distractor-attenuating strategy, it is not of central theoretical interest for the present review.

Third, and critically, it is not possible to obtain a pre-cue benefit on incongruent trials or mostly incongruent lists by using a distractor-enhancing strategy in task designs using a stimulus set that combines more than just two target and distractor feature values—that is, a design with *stimulus uncertainty* (Wühr & Kunde, 2008). For example, in a Stroop task that entails the stimuli BLUE, RED, YELLOW, and GREEN and their corresponding colors (i.e., a four-choice task), when pre-cued that the next trial will be incongruent, one cannot use the word to determine what the correct response will be because each word is equally predictive of the three incongruent response options (e.g., the word BLUE is equally predictive of a red, yellow and green response). Here, a pre-cue benefit emerges only if the biasing of attention in response to the pre-cue involves a nonspecific distractor-attenuating strategy (as in Fig. 3A–B). Critically, the literature we review in greatest depth below employs such task designs (three- or four-choice tasks), and pre-cue benefits observed under these conditions in principle map onto the real-world examples we offered at the start of this paper, which similarly involve situations in which the nature of distraction is variable and thus uncertain.

## Review of literature

Our review is divided into two sections. In the first section, we briefly review research that investigated pre-cue benefits within two-choice conflict tasks (see Table 1 for chronological list of studies included in review). These studies exclusively used the trial-level pre-cueing paradigm. Although findings from two-choice tasks are important and were highly influential in motivating subsequent investigations of pre-cue benefits, the findings speak less directly to the puzzle at hand given that a distractor-enhancing strategy may have produced the benefit on incongruent (as well as congruent) trials.

**Table 1:** Trial-level pre-cueing studies with two-choice conflict tasks using 100% valid pre-cues (chronologically ordered)

		*Task*	*Sample* *Size*	*Preparation* *Interval (ms)*	*Cue* *Type*	*Response Modality*	*Incongruent* *Pre-Cue Benefit (effect size)*	*Congruent* *Pre-Cue Benefit* *(effect size)*
Logan & Zbrodoff, 1982	Exp 1	Spatial Stroop	12	200/300/500/ 700/900/1,100	Symbolic	Manual	✓	✓
	Exp 2	Spatial Stroop	12	200/300/500/ 700/900/1,100	Symbolic	Manual	✓	✓
Aarts et al., 2008^*^	Exp 1	Arrow-word Stroop	12	2,600 to 7,600	Symbolic	Manual	✖	✓
Wühr & Kunde, 2008	Exp 1	Spatial Simon	40	1,200	Semantic	Manual	✓ (*d* = 0.39)	✓ (*d* = 0.49)
Correa et al., 2009	Exp 1	Arrow flanker	19	1,100 or 1,600	Symbolic	Manual	✓ (*d* = 0.52)	✓
Bugg & Smallwood, 2016^**^	Exp 2	Color-word Stroop	24	600/1,350/2,100	Semantic	Vocal	✓ for all CSI (*d* ≥ 1.32)	✓ for all CSI (*d* ≥ 2.48)
Chiew & Braver, 2016^*^	Exp 1	Arrow flanker	24	800	Symbolic	Manual	✓ for reward trials (*d* = 0.84)	✖ inverted for reward trials (*d* = 0.44)
	Exp 2	Arrow flanker	24	2,000	Symbolic	Manual	✓ for early reward	✓ for late reward

*Note.* Preparation Interval refers to the amount of time participants had to prepare in response to the pre-cue, that is, the total time between the onset of the pre-cue and the onset of the stimulus. In the Incongruent Pre-Cue Benefit and Congruent Pre-Cue Benefit columns, a checkmark indicates that performance in the cued condition was significantly faster than the uncued condition for incongruent trials or congruent trials, respectively. An ✖ indicates that a significant pre-cue benefit was not found. References to “inverted” mean that the effect was significant but in the reversed direction (i.e., a pre-cue cost instead of a pre-cue benefit). Effect sizes are provided for both significant and null findings, reported as Cohen’s *d* (*d*_z_). These values were converted from the original test statistics (e.g., *F*- or *t*-values) when available. An effect size is omitted if the necessary statistics for conversion were not reported in the source publication

* In the Incongruent Pre-Cue Benefit and Congruent Pre-Cue Benefit columns, a checkmark indicates that there was a pre-cue benefit (i.e., difference between informative and uninformative) for interference (incongruent-neutral) and/or facilitation (neutral-congruent) effects, respectively, which were the outcomes reported in these two studies

** Although this experiment used a four-choice task, the paradigm was designed to mimic the structure of a two-choice task. Each word appeared only with its congruent color and one other incongruent color (e.g., YELLOW appeared in yellow or blue ink, and never in red or green ink), which allowed participants to adopt a response prediction strategy on incongruent trials

Therefore, in the second section, we provide an in-depth review of research that has investigated pre-cue benefits under conditions of stimulus uncertainty, in three- and four-choice conflict tasks. This research speaks directly to the puzzle at hand and the question of primary interest regarding people’s ability to use pre-cues to bias attention in anticipation of distraction (i.e., use of a distractor-attenuating strategy, which—as a reminder—may involve down-weighting the task-irrelevant dimension and/or amplifying attention to the task-relevant dimension). In this section, we review findings from the trial-level paradigm separately from the list-level paradigm (see Tables 2 and 3, respectively, for chronological lists of studies included in review).

**Table 2:** Trial-level pre-cueing studies with three- or four-choice conflict tasks using 100% valid pre-cues (chronologically ordered)

		*Task*	*Sample* *Size*	*Preparation* *Interval (*ms*)*	*Cue Type*	*Response Modality*	*Incongruent Pre-Cue Benefit (effect size)*	*Congruent Pre-Cue Benefit (effect size)*
Wühr & Kunde, 2008	Exp 2	Spatial Simon	40	1,200	Symbolic	Manual	✖ inverted (*d* = 0.84)	✔ (*d* = 0.95)
	Exp 3	Spatial Simon	32	2,500	Symbolic	Manual	✖ inverted (*d* = 0. 75)	✔ (*d* = 0.28)
	Exp 4	Spatial Simon	28	1,200	Symbolic	Manual	✖	NA
Goldfarb & Henik, 2013	Exp 1	Color-word Stroop	16	1,500 or 2,500	Symbolic	Manual	✖	NA
	Exp 2	Color-word Stroop	32	1,500 or 2,500	Symbolic	Manual	✔ for neutral list (*d* = 0.97)	NA
Bugg & Smallwood, 2016	Exp 1	Color-word Stroop	18	600/1,350/ 2,100	Semantic	Vocal	✔ for 2100 ms (*d* = 0.92)	✔ for all CSI (*d* ≥ 1.06)
	Exp 4	Color-word Stroop	24	600/1,100/ 1,600/ 2,100	Semantic	Vocal	✔ for 2100 ms (*d* = 1.28)	✔ for all CSI (*d* ≥ 1.24)
Jiménez et al., 2021^*^	Exp 1	Color-word Stroop	32	750	Previous trial	Manual	✖ (2^nd^ half experiment: *d* = 0.28)	✖
	Exp 2	Color-word Stroop	32	1,750	Previous trial	Manual	✖ (2^nd^ half experiment: *d* = 0.06)	✖
	Exp 3	Color-word Stroop	24	750	Previous trial	Manual	✖ (final block: *d* = 0.38)	✖
	Exp 4	Color-word Stroop	24	1,750	Previous trial	Manual	✖ (final block: *d* = 0.10)	✖
	Exp 5	Color-word Stroop	32	750	Previous trial and symbolic	Manual	✖ (2^nd^ half experiment: *d* = 0.08)	✖
	Exp 6	Color-word Stroop	24	750	Symbolic	Manual	✖ (final block: *d* = 0.30)	✖
	Exp 7	Color-word Stroop	24	750	Symbolic	Vocal	✔ (final block: *d* = 0.48)	✔
	Exp 8	Color-word Stroop	24	750	Previous trial	Vocal	✖ inverted (final block: *d* = 0.23)	✖
	Exp 9	Color-word Stroop	24	750	Symbolic	Manual	✖ inverted (final block: *d* = 0.38)	✖ inverted
	Exp 10	Color-word Stroop	24	750	Symbolic	Vocal	✖ (final block: *d* = 0.22)	✔
Jiménez et al., 2024^*, **^	Exp 3	Color-word Stroop	24	750	Previous trial	Vocal	✖	✖
	Exp 4	Color-word Stroop	48	750	Previous trial	Vocal	✖	✖
Huang & Egner, in press	Exp 2	Spatial Stroop	39	2,000	Symbolic	Manual	✖	✖
	Exp 3a	Spatial Stroop	32	2,000	Symbolic	Manual	✖	✖
	Exp 3b	Spatial Stroop	30	2,000	Symbolic	Manual	✖	✖
	Exp 4	Spatial Stroop	32	2,000	Symbolic	Manual	✖ (*d* = 0.19)	✔ (*d* = 0.62)
	Exp 5	Spatial Stroop	30	2,000	Symbolic	Manual	✖	✖
Nuño & Bugg, 2025	Exp 1	Arrow flanker	56	600/1,350/ 2,100	Semantic	Vocal	✖	✖
Nuño & Bugg, 2026	Exp 1	Color-word Stroop	43	1,100	Semantic	Vocal	✔ for reward (*d =* 0.28) and no-reward (*d =* 0.18)	✔ for baseline (*d =* 0.68), no-reward (*d =* 0.71), and reward (*d =* 0.94)
	Exp 2	Color-word Stroop	47	1,100	Semantic	Vocal	✔ for reward (*d =* 0.16) and no-reward (*d =* 0.18)	✔ for baseline (*d =* 0.67), no-reward (*d =* 0.59), and reward (*d =* 0.40)
	Exp 3	Color-word Stroop	65	1,100	Semantic	Vocal	✔ for baseline (*d =* 0.32) and reward (*d =* 0.19)	✔ for baseline (*d =* 0.96), no-reward (*d =* 0.84), and reward (*d =* 0.94)

*Note. *Preparation Interval refers to the amount of time participants had to prepare in response to the pre-cue, that is, the total time between the onset of the pre-cue and the onset of the stimulus. In the Incongruent Pre-Cue Benefit and Congruent Pre-Cue Benefit columns, a checkmark indicates that performance in the cued condition was significantly faster than the uncued condition for incongruent trials or congruent trials, respectively. An ✖ indicates that a significant pre-cue benefit was not found. References to “inverted” mean that the effect was significant but in the reversed direction (i.e., a pre-cue cost instead of a pre-cue benefit). NA = not applicable because the experiment did not include congruent trials. Effect sizes are provided for both significant and null findings, reported as Cohen’s *d* (*d*_z_). These values were converted from the original test statistics (e.g., *F*- or *t*-values) when available. An effect size is omitted if the necessary statistics for conversion were not reported in the source publication

* For previous trial pre-cues, as used by Jiménez et al. (2021, 2024), the preparation time comprises both the intertrial interval, as listed in the table, and the participants’ response time on the previous trial, which is not included in the table

** Pre-cueing effects were also examined in two additional experiments (Experiments 1 and 2) within this study, but the authors concluded that the effects could not be clearly interpreted because of a confound (see text for details) and thus these studies were excluded from the review

**Table 3:** List-level pre-cueing studies with four-choice conflict tasks using 100% valid pre-cues (chronologically ordered)

		*Task*	*Sample Size*	*Cue Type*	*Response Modality*	*MI Pre-Cue Benefit (effect size)*	*MC Pre-Cue Cost (effect size)*
Bugg et al., 2015	Exp 1	Color-word Stroop	22	Semantic	Vocal	✖	✔ for list (*d* = 0.48)
	Exp 2	Color-word Stroop	20	Semantic	Vocal	✖	✔ for list (*d* = 0.77) and first trial (*d* = 0.56)
	Exp 3	Color-word Stroop	22	Semantic	Vocal	✖	✔ for list (*d* = 0.75) and first trial (*d* = 0.59)
	Exp 4	Color-word Stroop	48	Semantic	Vocal	✔ for first trial incentive (*d* = 0.31)	✔ for list (*d* = 0.43) and first trial (*d* = 0.25)
Bugg & Diede, 2018	Exp 1	Color-word Stroop	61	Semantic	Vocal	✖ (*d* = 0.14)	✖ (*d* = 0.19)
	Exp 2	Color-word Stroop	60	Semantic	Vocal	✖ (*d* = 0.12)	✔ when combined with Exp 1 (*d* = 0.22)
	Exp 3	Color-word Stroop	60	Semantic	Vocal	✖	✔ (*d* = 0.37)
Suh et al., 2022	Exp 1	Color-word Stroop	64	Semantic	Manual	✖	✔ globally low conflict block (*d* = 0.72), PC70 lists (*d* = 0.40), PC90 lists (*d* = 0.56)
	Exp 2	Color-word Stroop	48	Semantic	Manual	✖	✖
Nuño et al., 2025	Exp 1a	Color-word Stroop	84	Semantic	Vocal	✖	✔ for list (*d* = 0.40) and first trial (*d* = 0.25)
	Exp 1b	Color-word Stroop	86	Semantic	Vocal	✖	✔ for list (*d* = 0.68) and first trial (*d* = 0.64)
	Exp 2	Color-word Stroop	96	Semantic	Vocal	✔ for first trial (d = 0.29)	✔ for list (*d* = 0.60) and first trial (*d* = 0.28)
Nuño & Bugg, 2025	Exp 2	Arrow flanker	58	Semantic	Manual	✖	✔ for list (*d* = 0.27)
	Exp3	Arrow flanker	58	Semantic	Manual	✖	✖
	Exp 4	Arrow flanker	38	Semantic	Manual	✖	✖

*Note*. MI = mostly incongruent lists; MC = mostly congruent lists; In the MI Pre-Cue Benefit and MC Pre-Cue Cost columns, a checkmark indicates that the congruency effect was significantly smaller or larger, respectively, in the cued condition compared with the uncued condition. An ✖ indicates that a significant pre-cue benefit was not found. Effect sizes are provided for both significant and null findings, reported as Cohen’s *d* (*d*_*z*_). These values were converted from the original test statistics (e.g., *F*- or *t*-values) when available. An effect size is omitted if the necessary statistics for conversion were not reported in the source publication

In addition to focusing most of our review on studies that have utilized three- and four-choice conflict tasks, please also note the following: One, as previously mentioned, our review excludes studies that did not use 100% valid pre-cues because it is most puzzling not to observe pre-cue benefits for pre-cues that are accurate. Two, most of the pre-cueing literature focuses on response times rather than error rates as the main dependent variable, and we will do the same, only referencing error rate findings if they contradict response time findings or are otherwise notable. Three, our review largely does not detail findings pertaining to congruent trials or mostly congruent lists because we are most interested in how people use pre-cues to cope with expected distraction via a distractor-attenuating strategy (though we will still mention if congruent trials were included in a study since that is an important design feature). Pre-cue benefits on congruent trials and pre-cue costs in mostly congruent lists have been routinely observed, with a few exceptions, suggesting people generally use pre-cues to engage distractor-enhancing strategies (as detailed in Tables 1, 2, and 3). Thus, the reader can assume these effects were evidenced in the studies we review, unless otherwise noted. Accordingly, the reader also should assume that references to “pre-cue benefits” or “effects of pre-cues” within the review refer to benefits or effects observed on incongruent trials or mostly incongruent lists.

### Research investigating pre-cue benefits in two-choice tasks

In the seminal pre-cueing study, Logan and Zbrodoff (1982) cued congruent (e.g., ABOVE presented above fixation) and incongruent (e.g., ABOVE presented below fixation) trials in a manual, spatial Stroop task using symbolic pre-cues, and included an uninformative pre-cue (uncued) condition. The participants’ goal was to respond to the word and not its location. Regardless of whether pre-cues were randomly intermixed (Experiment 1) or blocked (Experiment 2), a pre-cue benefit was found for incongruent trials. Subsequent studies demonstrated the stability and generalizability of pre-cue benefits on incongruent trials in two-choice designs. One study used symbolic pre-cues in the arrow-based flanker task in which participants responded to the direction of the center arrow (with a left or right key press) while ignoring flanking arrows (Correa et al., 2009). Another study used semantic pre-cues in a Simon task in which participants responded to the color of a filled square while ignoring its location by pressing a left or right response key (Wühr & Kunde, 2008). Collectively, these early findings strongly supported the conclusion that participants utilize 100% valid pre-cues to enhance performance on incongruent trials when a two-choice design is used, regardless of cue type and conflict task. One possible exception is a study that employed an arrow-word Stroop task, where participants responded to direction words (left, right) placed inside left- or right-pointing arrows, or a rectangular box serving as a neutral condition (Aarts et al., 2008). The authors did not report whether there was a pre-cue benefit for incongruent trials (i.e., faster responses when cued compared with uncued). Rather they reported that Stroop interference was halved but not significantly reduced in the cued compared with uncued condition (*p* = .079)

More recently, Chiew and Braver (2016) utilized a two-choice arrow flanker task with symbolic pre-cues signaling a congruent, neutral (flankers did not correspond to a response key), or incongruent trial and compared performance to an uncued condition. A unique component of their study is that they examined the role of motivation in pre-cue use by additionally presenting an incentive pre-cue, signaling whether a reward could be earned for good performance. They found that the congruency pre-cues reduced interference on reward trials but not on no-reward trials, when the congruency and incentive pre-cues were presented concurrently (Experiment 1) and when the incentive cue preceded the congruency pre-cue (Experiment 2). Given the consistent pre-cue use observed on incongruent trials in prior two-choice studies without incentives including in the flanker task (Correa et al., 2009; Logan & Zbrodoff, 1982; Wühr & Kunde, 2008), it is surprising that this pattern was not found on no-reward trials. However, as the authors noted, this may reflect the devaluation of congruency pre-cues on no-reward trials (e.g., participants see the trials as a chance to take a break).

### Research investigating pre-cue benefits in three- and four-choice tasks

#### Trial-level pre-cueing

The puzzle of profitless pre-cues has its roots in the Wühr and Kunde (2008) study mentioned above (“Research Investigating Pre-Cue Benefits in Two-Choice Tasks”). In stark contrast to the reliable pre-cue benefits observed on incongruent trials within two-choice tasks, they did not find a pre-cue benefit in a three-choice Simon task while otherwise holding constant the methodology from their two-choice experiment. That is, there was no longer evidence that participants benefited from knowing in advance that the next trial would be incompatible. Additional experiments showed that this was true even when participants were given almost double the time to prepare in response to the pre-cue, and when congruent trials were removed from the design so that attending to the irrelevant dimension was never beneficial (which theoretically could have made it harder for participants to ignore the irrelevant dimension when a pre-cue signaling an incongruent trial appeared). Critically, the lack of a pre-cue benefit on incongruent trials in the three-choice design suggests participants did not employ a distractor-attenuating strategy in response to the pre-cues. Indeed, the findings raised the possibility that pre-cue benefits on incongruent trials may be limited to two-choice designs that enable participants to use a distractor-enhancing strategy.

Several studies that followed Wühr and Kunde (2008) examined pre-cue benefits in four-choice tasks and found only limited evidence for pre-cue benefits on incongruent trials that would be consistent with use of a distractor-attenuating strategy. We will review these studies chronologically. Using a manual, color-word Stroop task, Goldfarb and Henik (2013) used a task design with no congruent trials in Experiment 1, instead presenting incongruent (50%) and neutral (50%) trials. Symbolic pre-cues were used and intermixed with an uninformative pre-cue. Consistent with Wühr and Kunde, there were no pre-cue benefits (on either trial type). They reasoned that the lack of pre-cue benefits may have been attributable to already heightened proactive control in this congruent trial-free task design, such that the pre-cues were redundant with the attentional bias already induced by the list composition (i.e., control was already heightened, such that the congruency effect was already near floor). Therefore, in Experiment 2, they examined a mostly neutral list, in addition to a mostly incongruent list. Critically, they found a pre-cue benefit for incongruent trials that was selective to the mostly neutral list, consistent with the hypothesis that only in this list was there room for participants to use the pre-cues to heighten control beyond the baseline level induced by experience within the list. A pre-cue benefit was not found for neutral trials in either list. The finding of a pre-cue benefit for incongruent trials in this four-choice design was the first of its kind, and supported the possibility that people can adjust attention in a nonspecific, distractor-attenuating fashion to minimize distraction when anticipated (i.e., on-demand and in response to the pre-cue).

Also using the color-word Stroop task, albeit a vocal response version, Bugg and Smallwood (2016) investigated pre-cue benefits in a four-choice task. Unlike Goldfarb and Henik (2013), Bugg and Smallwood employed conditions that were 50% congruent as in the initial two-choice pre-cueing studies and used semantic pre-cues to signal congruent and incongruent trials, and a string of *X*s as the uninformative pre-cue. Importantly, a cue-to-stimulus (CSI) manipulation was included, with three levels (600 ms, 1,350 ms, 2,100 ms) with the authors reasoning that pre-cue benefits should be largest for incongruent trials at the longest CSI, given temporal constraints on proactive control (e.g., Braver et al., 2007; Meiran, 1996). In Experiment 1, the informative and uninformative pre-cues were presented in distinct blocks, and within each block there was a distinct sub-block for each CSI. The key finding was a significant pre-cue benefit only at the longest CSI for incongruent trials. This finding stood in stark contrast to the key finding from their second experiment, which also used a four-choice task, but modified the design so that each word could appear with its congruent color and only one incongruent color, thereby mimicking a two-choice design. Critically, this enabled participants to use the distractor-enhancing strategy of response prediction, and significant pre-cue benefits were observed at all three CSIs for incongruent trials.

Taken together, the findings of Bugg and Smallwood’s (2016) first two experiments suggested that preparation time was consequential selectively in the condition in which a distractor-attenuating strategy was needed to produce a pre-cue benefit. In Experiment 4, the authors sought to reproduce the pre-cue benefit for incongruent trials observed in Experiment 1, given the theoretical significance in providing an additional piece of evidence in support of willed attentional biasing and its dependence on ample preparation time. They randomly intermixed four CSIs (600 ms, 1,100 ms, 1,600 ms, 2,100 ms) within the cued and uncued blocks such that the amount of time available to prepare was not predictable from trial-to-trial. Again, a significant pre-cue benefit was observed for incongruent trials only at the longest CSI. The findings of Experiments 1 and 4 thus provided converging evidence for pre-cue benefits on incongruent trials in a vocal four-choice color word Stroop task, consistent with use of a nonspecific, distractor-attenuating strategy.

Five years later, in an extensive series of 10 experiments employing trial-level pre-cueing paradigms, Jiménez et al. (2021) investigated pre-cue benefits in a four-choice color-word Stroop task in which 50% of trials were congruent. Before describing these experiments, we wish to highlight three major takeaways from their study. First, they found very little evidence for pre-cue benefits, and interestingly, this was true for both incongruent trials, as well as congruent trials. Second, a pre-cue benefit was found for incongruent (and congruent) trials in only one experiment (Experiment 7); however, only the benefit for congruent trials was reproduced in a similar experiment (Experiment 10). Experiments 7 and 10 differed from the other experiments in that they employed a vocal Stroop task rather than a manual Stroop task. Moreover, Experiments 7 and 10 also used pre-cues that were presented independently of the Stroop task stimuli (i.e., during an intertrial interval [ITI]), whereas the other experiments (all manual) included pre-cues that followed this procedure or pre-cues that were a feature of the previous-trial Stroop stimulus (including one vocal). The findings led the authors to conclude that the on-demand heightening of control in response to pre-cues “is restricted to situations in which cues are presented alone, and where the task involves a nonarbitrary stimulus–response mapping (p. 1686).” We will next highlight the main methodology and findings of each experiment.

Experiments 1 and 2 used a manual Stroop task in which the target color (and associated response) from trial *n* − 1 was predictive of the congruency of trial *n.* One color (i.e., pre-cue) predicted a congruent trial, one color predicted an incongruent trial, and two colors served as controls (i.e., uncued [uninformative] condition), and these cues were randomly intermixed. Hereafter, we will refer to this as a *previous trial* pre-cue, meaning that the pre-cue is not presented on its own but is a feature of the stimulus on the prior trial. Participants were instructed about the predictive association between color and congruency (framed as how easy or difficult the next trial would be) and encouraged to use this information to respond more efficiently; in all experiments, a post-experimental questionnaire confirmed participants retained instructed associations. Experiments 1 and 2 were identical except that the ITI, which dictates preparation time, was 750 ms in Experiment 1 and 1,750 ms in Experiment 2.^6^ The key finding was that there was no pre-cue benefit on incongruent trials, nor surprisingly on congruent trials in either experiment.

Experiments 3 and 4 were similar to Experiments 1 and 2, except that now the cueing manipulation was blocked (rather than randomly intermixing the trial-level pre-cues corresponding to the cued and uncued conditions). Again, there was no pre-cue benefit on incongruent trials, or congruent trials. Interestingly, participants were *slower* overall in the cued compared with uncued condition (significantly so in Experiment 3 and nominally so in Experiment 4), raising the possibility that it was burdensome for participants to use the previous trial pre-cues, which required concurrent processing of the stimulus (in order to respond on trial *n* − 1) and the predictive information it conveyed about the next trial. To mitigate this dual demand, in Experiment 5, the pre-cue extended into the interval encompassed by the fixation point. Again, there were no pre-cue benefits for incongruent trials, or congruent trials.

Experiments 6 and 7 used the blocked design of Experiments 3 and 4. Previous trial pre-cues again were used but participants were not instructed about the color–congruency associations. Instead, instructions focused on the pre-cued information available during the ITI, namely a fixation appearing as one of three symbols (predicting a congruent or incongruent trial in the cued block or serving as an uninformative pre-cue in the uncued block). In Experiment 7, unlike Experiment 6 and all preceding experiments, participants responded vocally to the Stroop stimuli. Participants had 750 ms to prepare in response to the pre-cue. A 2,250-ms interval occurred after each response.^7^ The findings of Experiment 6 mirrored those of all preceding experiments showing no pre-cue benefits. However, in contrast, a significant pre-cue benefit was obtained in Experiment 7, on incongruent trials as well as congruent trials, the first evidence of a pre-cue benefit in this series of experiments.

Collectively, the lack of pre-cue benefits in any of the manual experiments paired with the presence of pre-cue benefits on the critical, incongruent trials in Experiment 7 led the authors to ask whether use of a vocal Stroop task was the key to finding pre-cue benefits. Thus, in Experiment 8, they reconducted Experiment 3 but used a vocal Stroop task. There was no pre-cue benefit. These findings suggest that use of a vocal Stroop task alone is insufficient for producing pre-cue benefits (see also Goldfarb & Henik, 2013, for evidence of pre-cue benefits on incongruent trials in a four-choice manual Stroop task, suggesting use of a vocal task is not necessary). Rather, the nature of the pre-cue (previous trial vs. symbolic intertrial) seemed to be a crucial factor.

In two final experiments, the authors again utilized the procedures from Experiments 6 and 7, with Experiment 9 mapping onto the manual version and Experiment 10 the vocal version, but they removed the 2,250-ms delay inserted following the response to each Stroop stimulus and, uniquely in Experiment 10, they removed the color–congruency associations from the task. In Experiment 9, there were no pre-cue benefits. In Experiment 10, there was no pre-cue benefit for incongruent trials (but the congruent trial benefit was reproduced). There are at least two possible explanations for the contrasting findings. One is that the pre-cue benefit for incongruent trials in Experiment 7 was a Type 1 error. This seems plausible if we assume that 750 ms is not enough preparation time to produce pre-cue benefits on incongruent trials in a four-choice Stroop task (Bugg & Smallwood, 2016; see also Goldfarb & Henik, 2013, for pre-cue benefits observed on incongruent trials when participants had between 1,500 and 2,500 ms to prepare).

A second possibility is that design differences explain the contrasting findings. In Experiment 10, the authors removed the association between the color of trial *n* − 1 and the congruency of trial *n*. This association existed in Experiment 7, but participants were not told about it and did *not* learn it (as assessed postexperimentally). Accordingly, we doubt this difference explains the contrasting findings. However, in Experiment 10, the authors also eliminated the 2,250-ms interval between the Stroop response and the subsequent pre-cue. Because the interval occurred before the pre-cue, this did not change the amount of time participants had to prepare a distractor-attenuating strategy. However, the additional time may have enabled participants to deactivate the strategy (attentional bias) used on the preceding trial uniquely in Experiment 7 (akin to the deactivation of a task set, which benefits from an extended ITI). For example, if the preceding trial was congruent (and participants presumably used the pre-cue to read the word; i.e., a distractor-enhancing strategy) and the next trial was incongruent (requiring a distractor-attenuating strategy), having less time to deactivate the previously used strategy before receiving the incongruent pre-cue may have made it more difficult to reap the benefits of the pre-cue on incongruent trials. Applying similar reasoning, one might also expect the pre-cue benefit on congruent trials to be attenuated in Experiment 10 if the 2,250-ms interval was beneficial in this way. Indeed, that benefit was halved (61 ms in Experiment 7 to 30 ms in Experiment 10).

Extending this research, in four experiments Jiménez et al. (2024) examined how list composition (proportion of incongruent and congruent trials) affects pre-cueing effects. They adopted the methodology from Experiment 8 of Jiménez et al. (2021) which entailed a vocal version with previous trial pre-cues. As noted above, using this type of pre-cue may be quite effortful, and the authors questioned whether reducing the accumulated cost across trials by reducing the number of incongruent trials would reveal pre-cue benefits. Because a confound affected interpretation of the first two experiments, as the authors discussed, we only consider results from Experiments 3 and 4.^8^ In these experiments, all colors appeared equally frequently. In Experiment 3, one color was designated to be predictive of incongruent trials while the three other colors predicted congruent trials, thus creating a mostly (75%) congruent list. In Experiment 4, one color was predictive of incongruent trials and one color was predictive of congruent trials, while the other two colors predicted a neutral Stroop stimulus (a string of *X*s presented in one of the four colors). Incongruent trials thus occurred with the same frequency as in Experiment 3 (on 25% of trials). In neither experiment was there a pre-cue benefit for incongruent trials (or congruent trials). Collectively, the findings converge with Jiménez et al. (2021) in showing that participants do not utilize previous trial pre-cues.

One potential explanation for the limited evidence for pre-cue benefits in four-choice tasks compared with two-choice tasks is that use of a distractor-attenuating strategy is presumably more effortful than a distractor-enhancing strategy (i.e., response prediction), and thus potentially more dependent on motivation. To address the role of motivation, in an unpublished study, Nuño and Bugg (2026) examined whether incentivizing performance in a four-choice, vocal color-word Stroop task would motivate participants to use incongruent trial pre-cues in a condition in which pre-cue benefits have not been previously observed—namely, when a 1,100 ms CSI is employed (see Bugg & Smallwood, 2016). The design mirrored Bugg and Smallwood (2016, Experiment 1) except only the 1,100 ms CSI was employed and participants had the opportunity to earn a monetary bonus on reward trials if they were accurate and faster than in a baseline phase (during which rewards were not available on any trials). During the test phase, incentive cues ($$$ = reward trial and XXX = no-reward trial) were presented before congruency pre-cues on each trial. Experiments 1 and 2 were similar, except reward/no-reward trials were intermixed within a block or presented in separate blocks, respectively. There was not a pre-cue benefit on incongruent trials during the baseline phase, akin to Bugg and Smallwood (2016). In contrast, in both experiments, a pre-cue benefit was found for incongruent trials during the test phase; however, this effect was observed on reward and no-reward trials, suggesting some carry-over of motivational effects associated with the reward or the knowledge of a potential reward. In Experiment 3, reward availability was manipulated between subjects. Unexpectedly, a pre-cue benefit was observed in both groups in the baseline phase. Critically, only the rewarded group showed a significant pre-cue benefit on incongruent trials in the test phase, supporting a role for motivation in on-demand adjustments in control in response to pre-cues. However, the magnitude of pre-cue benefits did not differ between the reward and no-reward groups in Experiment 3, nor was a difference found between reward conditions in Experiments 1 or 2. The lack of a difference may be due to carry-over effects in Experiments 1 and 2, or use of a between-subjects design in Experiment 3. Alternatively, the effects of motivation may be subtle under conditions in which there is limited time to prepare.^9^

Finally, two very recent studies add further breadth to the lack of pre-cueing benefits across a wider variety of paradigms. First, in an unpublished four-choice flanker task study (Nuño & Bugg, 2025, Experiment 1), there was no evidence for pre-cue benefits on incongruent trials (or congruent trials) when using the same design as Bugg and Smallwood (2016) in which CSI was manipulated (2016, Experiment 1). The lack of a pre-cue benefit for the 2100 ms CSI is particularly surprising because that benefit was found for incongruent trials in the color-word Stroop task in two experiments in Bugg and Smallwood. Second, Huang and Egner (in press) conducted a series of experiments using a variant of the spatial Stroop task (Viviani et al., 2024) to directly test whether the lack of pre-cue benefits in manual color-word Stroop studies stemmed from the arbitrary nature of their response mappings. In this task, arrows appeared in four spatial positions (top-left, top-right, bottom-left, bottom-right) of a central square, and participants responded to the arrow’s pointing direction while ignoring its spatial location using spatially corresponding keys. This preserved the nonarbitrary response characteristics of vocal Stroop tasks while using manual responses. In five experiments using 100% valid symbolic pre-cues with 2000 ms preparation time, they manipulated ITIs (Experiments 3a and 3b: 1,000 ms and 2,250 ms), task difficulty through brief stimulus presentation (Experiment 4: 50 ms), and list composition (Experiment 5: 75% congruent trials). Despite robust congruency effects, no pre-cue benefits emerged for incongruent trials (or congruent trials, except in one experiment). Thus, the use of nonarbitrary stimulus–response mappings alone is insufficient for pre-cue benefits to emerge on incongruent trials.

#### Summary of key findings from trial-level pre-cueing paradigms

To take stock of the trial-level pre-cueing experiments reviewed in the above section, the following conclusions can be drawn. The evidence for pre-cue benefits on incongruent trials is mixed in three- and four-choice tasks, where there is stimulus uncertainty and participants must employ a distractor-attenuating strategy (i.e., down-weighting the task-irrelevant dimension and/or amplifying attention to the task-relevant dimension) to obtain pre-cue benefits. Starting with the positive evidence for pre-cue benefits on incongruent trials, there are seven experiments in which such benefits were observed (Bugg & Smallwood, 2016, Experiments 1 and 4; Goldfarb & Henik, 2013, Experiment 2; Jiménez et al., 2021, Experiment 7; Nuño and Bugg, 2026, Experiments 1, 2, and 3), all of which employed a color-word Stroop task, used 100% valid pre-cues (though they varied with respect to type of pre-cue), and gave participants at least 1,500 ms of preparation time, except Experiment 7 of Jiménez et al. (2021), which used a 750-ms CSI, and Nuño and Bugg (2026), which used a 1,100 ms CSI (paired with a monetary incentive manipulation). Additionally, all seven experiments involved vocal responses, except Goldfarb and Henik (2013).

In contrast to this positive evidence, there are multiple experiments within the same studies that used 100% valid pre-cues and did not observe pre-cue benefits on incongruent trials in the color-word Stroop task. All these experiments used a manual task except Jiménez et al. (2021, Experiment 10), which used a vocal task approximating their Experiment 7 where a benefit was found. There was no pre-cue benefit in Experiment 1 of Goldfarb and Henik (2013) who used lists comprising 50% incongruent and 50% neutral trials. Along with the findings from their second experiment showing no pre-cue benefits when a mostly incongruent list is used, these patterns may suggest that people may be more apt to use pre-cues when they are not in an environment in which control is already heightened in response to encountering conflict frequently. But, inconsistent with this suggestion, in two experiments, Jiménez et al. (2024) found no pre-cue benefits even in mostly congruent lists.

Eight additional experiments within Jiménez et al. (2021) did not yield a pre-cue benefit on incongruent trials. In five of the eight experiments, all of which used a manual task except one, symbolic, previous trial pre-cues were used (see also Jiménez et al., 2024, for two additional experiments using this type of pre-cue that yielded no evidence of a pre-cue benefit on incongruent trials). The consistent lack of benefits (including congruent trials in all five of these experiments) suggests that this type of pre-cue (i.e., previous trial) may be too effortful or difficult to use. Indeed, on congruent trials, participants simply could have used the distractor-enhancing strategy of attending to the word to perform faster in the cued than the uncued condition, a strategy that has yielded benefits in two-choice manual (Logan & Zbrodoff, 1982) and four-choice vocal (Bugg & Smallwood, 2016) Stroop tasks. The remaining three experiments within Jiménez et al. (2021) that did not yield a pre-cue benefit on incongruent trials also used a manual task, but with symbolic pre-cues presented during the fixation period. One possible explanation for null effects in these experiments is that pre-cue use generally may be more limited in manual tasks due to the additional load of maintaining the arbitrary stimulus–response mappings on top of the pre-cueing rules (i.e., which pre-cue is predictive of which trial type), as Jiménez et al. (2021) noted. However, broadly equivalent experiments involving nonarbitrary manual responses in a spatial Stroop task also failed to observe evidence for pre-cue benefits (Huang & Egner, in press), so the nature of the response mapping is unlikely to be the sole cause of null effects. Finally, the puzzling lack of pre-cue benefits on incongruent trials extends beyond some iterations of the color-word Stroop task to the Simon task (Wühr & Kunde, 2008), the flanker task (Nuño & Bugg, 2025), and the spatial Stroop task (Huang & Egner, in press), all of which were manual.

In a later section we develop a novel theoretical framework to account for and synthesize these mixed findings across trial-level pre-cueing experiments. Before doing so, we review studies examining pre-cueing effects in list-level paradigms.

#### List-level pre-cueing

The literature examining list-level pre-cueing effects is not as expansive as the trial-level literature, but it is still informative with respect to the puzzle at hand and uniquely speaks to the ability to engage a distractor-attenuating strategy in a more temporally extended (sustained) fashion. As a reminder, our review will focus primarily on pre-cue benefits in mostly incongruent lists (see Table 3 for a summary of findings from mostly incongruent and mostly congruent lists). All the reviewed experiments examining list-level pre-cueing have used four-choice tasks, enabling insights into people’s ability to engage a distractor-attenuating strategy in response to pre-cues signaling a mostly incongruent list.

In 2015, Bugg, et al. developed a novel list-level pre-cueing paradigm to examine whether people use advance information to adjust control on demand in a temporally extended way (beyond a single trial). They conducted four experiments using a four-choice vocal, color-word Stroop task. In Experiment 1, the cues 80% CONFLICTING and 80% MATCHING were presented in advance of mostly incongruent and mostly congruent lists, respectively (cued condition), while an uninformative pre-cue (?????) preceded these lists (50% of each list type) in the uncued condition. The three pre-cues were randomly intermixed. On each 10-trial list, the pre-cue was presented until the participant pressed a response key indicating they were prepared to begin the list. Of greatest interest was whether a pre-cue benefit (i.e., a reduction in the mean congruency effect) would be found for cued mostly incongruent lists compared with uncued mostly incongruent lists. No such benefit was observed, suggesting that knowing that the list would mostly comprise distracting trials did not lead to a heightening of control. Experiment 2 aimed to reproduce this finding and examine whether pre-cue benefits may be evident on the first trial following the pre-cue in a combined analysis of data from Experiments 1 and 2.^10^ The list-level analyses from Experiment 2 replicated Experiment 1 (i.e., no pre-cue benefit for mostly incongruent lists) and the findings from the first-trial analyses showed there also was no evidence of a more transient adjustment in response to the pre-cue signaling a mostly incongruent list.

Faced with these unexpected findings, in Experiments 3 and 4, Bugg et al. (2015) aimed to promote pre-cue use by adding time pressure (Experiment 3) or providing an incentive for good performance (Experiment 4). In Experiment 3, the authors reasoned that presenting the Stroop stimulus for only 100 ms would place a premium on preparation because, if unprepared, given the relatively faster processing of the irrelevant word than the relevant color, the word should detrimentally influence response selection. In this experiment, pre-cues signaled both the proportion congruency of the list and whether the list was speeded or not, for example 80% CONFLICTING/FASTER SPEED. Although the manipulation was effective in speeding responses, the list-level and first-trial analyses for the mostly incongruent lists closely mirrored Experiment 1, except there was a hint of a pre-cue benefit (*p* = .052) on the first trial selectively in speeded lists (this analysis may have been constrained by power, given the reduced observations when using only the first trial).

In Experiment 4 of Bugg et al. (2015), a point-based incentive manipulation was used with the goal of examining whether motivational constraints were limiting pre-cue use in the mostly incongruent lists. The incentive cues signaling the value of a given list (5 points or 50 points) were presented to participants after they indicated their preparedness in response to the proportion congruency pre-cue. Points were rewarded (or not) after each list based on how participants performed relative to a baseline phase. Participants were faster overall in the high-incentive than the low-incentive condition. At the list-level, the point-based incentive did not motivate participants to utilize the pre-cues in a temporally extended, sustained fashion. However, a pre-cue benefit was found when examining performance on the first trial of the lists. The congruency effect was reduced on the first trial for cued mostly incongruent lists (relative to uncued mostly incongruent lists) uniquely in the high-incentive condition. Critically, this represented the first evidence of a pre-cue benefit in the list-level paradigm. Given that the benefit was limited to the first position and only observed in the high-incentive condition, the findings suggested that participants can bias attention in a distractor-attenuating fashion in response to list-level pre-cues, though only transiently, and this biasing of attention may be motivation dependent (see Nuño and Bugg, 2026, for evidence within the trial-level paradigm).

Subsequent research aimed to address other potential explanations for the minimal evidence of pre-cue benefits in mostly incongruent lists. One potential explanation pertains to use of a within-subjects design in which the cued and uncued conditions are randomly intermixed within the experimental block. According to the *bleed-over of awareness hypothesis* (Bugg & Diede, 2018), because of the presence of cued lists, participants may become aware that lists vary in proportion congruency and attempt to detect the list type and adjust control accordingly when presented with an uninformative pre-cue (e.g., if the first couple of trials of an uncued list are incongruent, they ramp up control). Such bleed-over would reduce the difference in performance between cued and uncued lists, potentially precluding observation of pre-cueing effects. Of course, the presence of consistent pre-cue costs in mostly congruent lists (Bugg et al., 2015) presents a challenge for this view, but nonetheless Bugg and Diede (2018) investigated this possibility by utilizing a between-subjects manipulation of cueing in a vocal Stroop task. Participants in the cued condition never encountered uninformative pre-cues (and vice versa); otherwise, the design was similar to Bugg et al. (2015). The key finding was that the patterns observed previously in the within-subjects design of Bugg et al. (2015) were reproduced in a combined analysis of Experiments 1 and 2, including most importantly the lack of a pre-cue benefit in mostly incongruent lists. This finding was reproduced in Experiment 3. Additionally, Bugg and Diede found that participants’ awareness of list types in uncued conditions was unrelated to their performance. Overall, the findings suggested that the lack of a pre-cue benefit in mostly incongruent lists could not be attributed to a bleed-over of awareness.

A second potential explanation, which we refer to as the *redundancy hypothesis* (akin to the hypothesis of Goldfarb & Henik, 2013, within the trial-level pre-cueing literature), posits that control may already be at a functional ceiling resulting in floor-level congruency effects based on experience alone in mostly incongruent lists, such that no difference is observed between the cued and uncued mostly incongruent lists (i.e., there is no pre-cue benefit). On the one hand, the evidence for a pre-cue benefit on the first trial of mostly incongruent lists in the high-incentive condition of Experiment 4 of Bugg et al. (2015) (and the suggestive evidence in the speeded condition of Experiment 3) might be taken as evidence against this hypothesis. On the other hand, it may reinforce the hypothesis, since it can be argued that any experience-dependent adjustments would not yet be operative when responding to the first trial.

Suh et al. (2022) attempted to gain traction on the redundancy hypothesis by manipulating the global context in which cued and uncued lists were encountered in a pre-cued lists paradigm with manual responses. In one condition, the global probability of encountering incongruent trials was 66% (high-conflict global context) whereas in the other condition it was 34% (low-conflict global context). They achieved these probabilities while still being able to compare lists that were otherwise matched across the two global context conditions by including filler lists. In the high-conflict global context, filler lists were 90% incongruent, but in the low-conflict global context, they were 10% incongruent. Critically, there were three test lists: one that was mostly incongruent, one that was 50% congruent and one that was mostly congruent, and in each global context participants encountered cued and uncued versions of the three test lists randomly intermixed with the filler lists. The key question was whether pre-cue benefits would be observed for the mostly incongruent lists if the lists were encountered in a global context in which conflict was encountered infrequently, such that control might not already be maximally heightened. The answer was no. Again, there was no evidence of a pre-cue benefit for mostly incongruent lists (nor was there a benefit for 50% congruent lists). Although of less theoretical interest, it is notable that the typical pre-cue cost (larger congruency effect on cued than uncued mostly congruent lists) indicative of a relaxation of control (distractor-enhancing strategy), was modulated by the global context. This cost was found in the low- but not high-conflict global context and this pattern was replicated in a second experiment. The collective evidence from Suh et al. suggests that global probabilities of conflict affect pre-cue use, but only by limiting use of mostly congruent pre-cues in high-conflict contexts and not by amplifying use of mostly incongruent pre-cues in low-conflict contexts. Again, the findings did not support the redundancy hypothesis (see also Jiménez et al., 2024, for findings from trial-level pre-cueing paradigms that do not align with the redundancy hypothesis, although in their case they questioned whether reducing overall effort associated with pre-cue use by presenting incongruent trials infrequently in mostly congruent lists might reveal pre-cue benefits).

To take stock, findings from multiple experiments provided no evidence demonstrating use of pre-cues in mostly incongruent lists when examining differences in list-level congruency effects, calling into question whether people can sustain an attentional bias to minimize the effects of anticipated distraction. Furthermore, only one experiment revealed evidence that such a bias can be transiently executed in response to a pre-cue signaling a mostly incongruent list and that evidence stemmed from the first trial analyses in Bugg et al. (2015, Experiment 4)—namely, in the high-incentive condition. Suh and Bugg (2021) reported a reanalysis of the experiments from Bugg et al. (2015) using generalized linear mixed models (GLM) examining changes associated with the pre-cues on a trial-by-trial basis (by entering trial positions 1–10 in a model along with cueing and congruency). This allowed for investigation of pre-cueing effects on a finer scale than the entire list and a more extended scale than the first trial, as well as examination of how pre-cueing interacts with experience-driven adjustments in control. For mostly incongruent lists, perhaps the most striking finding was that in the speeded condition of Experiment 3 and high-incentive condition of Experiment 4, GLM revealed a pre-cue benefit extending beyond the first trial, indicating more sustained use of the pre-cues than initially thought based on the mean, list-level analysis of variance (ANOVA) on congruency effects in Bugg et al. (2015; though this pattern was also unexpectedly found in the unspeeded condition of Experiment 3). In addition, and interestingly, in the speeded condition (but not the high-incentive condition), the slope representing the congruency effect across trial positions was not significant when the list was pre-cued, but it was when the list was uncued. This suggests the typical experience-based adjustments in control that accumulate across mostly incongruent lists were not evident when control was heightened via the pre-cue in the speeded condition. In sum, the findings of Suh and Bugg pointed to new methods for investigating the influence of list-level pre-cues and the interactivity of pre-cueing with experience-based adjustments in control.

Thus far, in all experiments reviewed in this section, the question of whether people use list-level pre-cues to heighten control was examined by investigating performance in cued mostly incongruent lists. Drawing on the redundancy hypothesis, Nuño et al. (2025) adopted a different approach by asking whether a heightening of control instead might be observable in mostly congruent lists. In Experiment 1a participants were told that incongruent trials would be harder when they occurred rarely (were unexpected) and therefore they should focus on the goal of color naming when a mostly congruent pre-cue was presented. In contrast, they were told that incongruent trials would be easier following a mostly incongruent pre-cue, since such trials would be expected. Experiment 1b served as a comparison condition, using instructions akin to the original list-level pre-cueing study (Bugg et al., 2015) where the mostly congruent lists were highlighted as easy and the mostly incongruent lists as difficult. The key findings emerged in both the list level and first trial analyses: In Experiment 1a, there was a cue-induced cost suggesting that, despite the instructions, there was still evidence for a relaxation of control in response to the mostly congruent pre-cue. This was also true (as expected) in Experiment 1b. However, and importantly, the cue-induced cost was attenuated in Experiment 1a compared with Experiment 1b. This finding provides modest evidence for a heightening of control in cued mostly congruent lists when participants expect the rare incongruent trials to be disruptive, providing some support for the redundancy hypothesis. However, the heightening was not strong enough to overcome the relaxation of control that accompanies mostly congruent lists, and thus there was not a pre-cue benefit (reduced congruency effect in cued mostly congruent lists compared with uncued mostly congruent lists). In both experiments, pre-cue benefits in mostly incongruent lists were not observed.

Given that there was not a pre-cue benefit, in Experiment 2, Nuño et al. (2025) attempted to facilitate use of a distractor-attenuating strategy in the mostly congruent lists by having participants form an implementation intention (Gollwitzer, 1999), an if-then statement that helps people attain goals, such as those related to health and cognition (see, e.g., Adriaanse et al., 2011; Chen et al., 2016), including cognitive control (Cohen et al., 2008). Participants wrote down “When I see the mostly congruent pre-cue, I will focus hard on naming the ink color for each of the 10 trials after the pre-cue” and imagined seeing the pre-cue and implementing the strategy. The idea is that the behavior (“focusing hard …”) should be efficiently and automatically implemented when the critical cue is detected (Gollwitzer, 1999; Wieber et al., 2015). As in Experiments 1a and 1b, participants again relaxed control (there was a pre-cue cost) in the mostly congruent lists; relative to Experiment 1b, the cost was reduced, suggesting some heightening of control with use of implementation intentions in Experiment 2 but only in the first-trial analyses (i.e., only transiently) and not at the list level. In sum, the findings suggested that list composition plays some role in observing evidence for a heightening of control, but that role is modest and other factors contribute to the minimal evidence for pre-cue benefits in mostly incongruent lists. Unexpectedly but of interest, in Experiment 2 there was a pre-cue benefit for mostly incongruent lists in the first-trial analyses. This may suggest participants indiscriminately applied the implementation intention to both pre-cues (mostly congruent and mostly incongruent). To the extent that implementation intentions automatize behavior, as has been posited (Gollwitzer, 1999), the finding bolsters the view that pre-cue benefits in mostly incongruent lists (at least on the first trial) may be observed when motivational constraints are removed (see also incentive experiment of Bugg et al., 2015).

One important limitation of the above studies examining list-level pre-cueing is that all of them utilized the color-word Stroop task, and thus conclusions are tentative with respect to other conflict paradigms (flanker, Simon). In a set of unpublished experiments (Nuño & Bugg, 2025), the list-level pre-cueing paradigm was applied to a four-choice flanker task, with Experiment 2 implementing the design and procedure of Bugg et al. (2015, Experiment 1). The findings reproduced those of Bugg et al., most critically observing no evidence of a pre-cue benefit in mostly incongruent lists at the list level or in the first-trial analyses. However, mean error rates were significantly reduced in cued than uncued mostly incongruent lists—providing evidence of a heightening of control. This finding raised the possibility that error rate might be more sensitive to pre-cue use in mostly incongruent lists than response time. Hence, in Experiment 3 a response deadline was implemented (485 ms) to increase task difficulty, with the goal of shifting effects into error rate (cf. Jacoby et al., 2003). Although error rates were substantially increased, there was no evidence of a pre-cue benefit in mostly incongruent lists (or a pre-cue cost in mostly congruent lists).

In a final experiment (Experiment 4), Nuño and Bugg (2025) implemented the design and procedure from Bugg et al. (2015, Experiment 3) where stimuli were presented only briefly (100 ms) to encourage participants to prepare in response to the pre-cue. Recall that Bugg et al. found no pre-cue benefit at the list level in mostly incongruent lists when stimulus presentation was speeded, but the first-trial analysis revealed a marginal effect. When using the flanker task, the same pattern of results emerged, indicating no pre-cue benefits. The initial evidence from this study suggests that the lack of pre-cue benefits in the list-level pre-cueing paradigm may not be specific to the Stroop task, but instead generalizes to the flanker task, highlighting that the mechanisms underlying list-level pre-cue benefits may be consistent across these conflict tasks.

#### Summary of key findings from list-level pre-cueing paradigms

The preponderance of evidence clearly suggests people do not use pre-cues to heighten control on demand in list-level paradigms when frequent distraction is expected. Across multiple experiments, there was no evidence for a pre-cue benefit at the list-level in mostly incongruent lists, suggesting participants did not use a distractor-attenuating strategy (i.e., down-weighting the task-irrelevant dimension and/or amplifying attention to the task-relevant dimension). A few hypotheses were tested to explain the lack of a list-level pre-cue benefit (bleed-over of awareness, and redundancy), but for the large part the evidence did not support them. Another possible explanation is that although list-level pre-cues are 100% valid in signaling whether a *list* will be mostly incongruent, they provide probabilistic information on a trial-by-trial basis, which may deter pre-cue use.

On a positive note, there was evidence of a pre-cue benefit in mostly incongruent lists in one experiment that rewarded good performance with a point-based incentive (Bugg et al., 2015, Experiment 4), and unexpectedly in an experiment in which participants formed implementation intentions to focus on color naming in response to the pre-cue signaling a mostly congruent list (Nuño et al., 2025). These benefits were, however, restricted to the first trial within the list (but see Suh & Bugg, 2021, for GLM findings suggesting a more extended benefit). These first-trial findings lend support to the notion that people can bias attention in a transient fashion to minimize anticipated but uncertain distraction, so long as they are incentivized to do so (Bugg et al., 2015) or motivational constraints are otherwise overcome (Nuño et al., 2025).

## TEPID: A novel framework for understanding the puzzle of profitless pre-cues

One way of describing pre-cue use in the three- and four-choice trial-level and list-level pre-cueing literatures reviewed above is tepid, meaning that it is marked by a lack of force or enthusiasm. Taking inspiration from this observation, we propose the TEPID framework, which posits that **T**ask-related (or **E**xternal) factors and **P**erson-related (or **I**nternal) factors, alongside limitations of pre-cueing **D**esigns, dictate pre-cue benefits (or the absence thereof). Although the notion that task (external) and person (internal) factors, as well as design parameters, may account for variation in a given psychological phenomenon is not new, this multifaceted theoretical framework is novel in integrating key findings from the literature to explain why pre-cue use has been tepid and identifying conditions that are predicted to maximize evidence for pre-cue benefits, predictions that can be tested in future studies.

Before defining the multiple factors at play, we first ask the reader to consider what it might be like to participate in one of the four-choice pre-cueing experiments we described, such as Bugg and Smallwood (2016). Imagine that in addition to being instructed about the Stroop task (i.e., “name the color as quickly and accurately as possible while ignoring the word”) at the start of the experiment, you receive instructions about two, 100% valid pre-cues: CONFLICTING, which indicates an incongruent trial will follow, and MATCHING, which indicates a congruent trial will follow. You receive some practice trials before jumping into a test block, in which the two pre-cues are randomly intermixed. Imagine that the pre-cue CONFLICTING appears on screen. What would you do when it appears? One possibility is that you may not know what to do and thus you might do nothing. Alternatively, you may have an intuition about what to do (e.g., devise a strategy that maps onto the nonspecific distractor-attenuating mechanism), but you may not be able to engage such a strategy (at all or within the time afforded to process the pre-cue and prepare accordingly). In these cases, you may feel like you *should* be able to benefit from the pre-cues (aligning with the omnipresent intuitions many hold regarding people’s ability to focus attention on-demand), but your performance may suggest otherwise. On the other hand, imagine that you heed the instructions and when seeing CONFLICTING, you engage a strategy in anticipation of an incongruent stimulus that involves trying to “block out the word” or “focus harder on the color” so that you are not distracted by the word. If you imagine trying to use the pre-cues in these ways, it might strike you that it feels effortful to do so and, as such, may require time and motivation. Now, let us further assume that you are motivated and able to engage this strategy, but the Stroop stimulus appears shortly after the pre-cue and thus you feel like your preparation was incomplete (i.e., cut short). Would you continue to expend the effort on subsequent trials? What if you instead had ample time to make the preparatory adjustments (i.e., there is a longer delay between the pre-cue and the Stroop stimulus)? Now the effort may feel justifiable.

This exercise highlights how *task-related factors*, which are variables *external to the participant* like preparation time, and *person-related factors*, which are variables that are *internal to the participant* like ability and motivation, influence pre-cue use and accordingly, whether pre-cue benefits are observed. This example assumed that you (the participant) were motivated to use the pre-cues, but lack of motivation is likely a critical factor that limits pre-cue use alongside other factors highlighted (e.g., lack of sufficient time to prepare, and inability to translate the information offered by the pre-cue into an effective strategy), as we will discuss momentarily.

As a point of comparison, we also would like you to consider briefly the other conditions you would encounter if you were a participant in this experiment. Imagine how different it would be to encounter the pre-cue MATCHING, and the minimal time and effort you would need to benefit from this pre-cue (i.e., preparing a distractor-enhancing strategy of reading the word when the stimulus is presented). Now imagine what it might feel like when encountering the uninformative pre-cue XXXXXXXXX. Perhaps it would evoke uncertainty, and the 50% likelihood of an incongruent trial might lead you to prepare in the same way that you prepared for the pre-cue CONFLICTING. Alternatively, you might feel that this pre-cue is useless and thus you do not engage in any preparatory adjustments but instead use a purely reactive strategy. This is generally what researchers assume will happen, given that this condition is treated as a control condition for evaluating benefits of the informative pre-cues. (Note that this assumption will be examined in a later section, “Design Limitations”).

Bearing in mind this example, we will now describe the task (external) and person (internal) factors that we believe to be most critical in modulating pre-cue benefits in three- and four-choice task designs. Thereafter, we propose a two-phase model of how these factors influence pre-cue use and accordingly, pre-cue benefits. Then we consider limitations of pre-cueing designs that may also be contributing to the limited evidence for pre-cue benefits on incongruent trials and in mostly incongruent lists. The TEPID framework draws on findings from our literature review (including the contrasting pattern of pre-cue benefits on incongruent trials routinely found in the two-choice literature) and the broader cognitive control literature, in addition to novel theorizing to identify key factors and describe a model of pre-cue use, as well as high-priority targets for future research.

### Task (external) factors

**Fig. 4:**
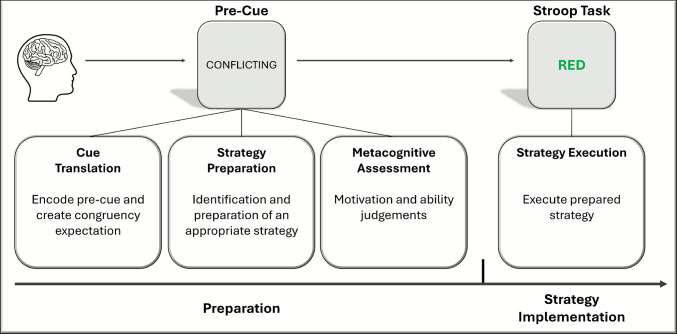
This figure illustrates the two sequential phases of pre-cue elicited cognitive control proposed by the TEPID framework. The preparation phase begins with pre-cue presentation, which triggers three interrelated processes: translating the cue (converting the cue into expectations about upcoming stimulus congruency/list proportion congruency), identifying and preparing an appropriate strategy, and performing a metacognitive assessment (evaluating the costs and benefits of engaging control mechanisms and one’s ability to engage the identified strategy). The subsequent strategy implementation phase depends on sufficient preparation time and participant’s motivation/ability to execute their chosen strategy. (Color figure online)

Our review of the literature, especially studies using trial-level pre-cueing paradigms, points to *preparation time* as a central task (external) factor. Preparation time is the length of time between the onset of the pre-cue and the presentation of the stimulus (see Tables 1 and 2 for the preparation times in reviewed literature). In all studies that observed pre-cue benefits on incongruent trials, participants had at least 1,500 ms to prepare (Bugg & Smallwood, 2016, Experiments 1 and 4; Goldfarb & Henik, Experiment 2, 2013), with two exceptions (Jiménez et al., 2021, Experiment 7; Nuño and Bugg, 2026). The findings of Bugg and Smallwood (2016), who directly contrasted CSIs ranging from 600 to 2,100 ms, demonstrated pre-cue benefits for incongruent trials only at the longest CSI, further reinforcing the importance of preparation time. Why does preparation time matter? Participants must engage in a variety of processes during the preparatory interval to gain a pre-cue benefit: cue identification, cue translation (inferring what the cue means), and construction of an appropriate strategy depending on the pre-cue and design type (see Fig. 4). In three- and four-choice task designs where it is necessary to engage a distractor-attenuating strategy to gain a pre-cue benefit, pre-cue benefits should depend on the time available to prepare (cf. Horváth, 2013), with larger benefits observed at longer CSIs (see Bugg & Smallwood, 2016, Experiment 2). However, there are two caveats. One, information conveyed by the pre-cue may be vulnerable to decay or interference if the CSI is too long (perhaps especially if the cue is not presented for the duration of the CSI but only briefly at the beginning of the preparatory interval). Two, the precise relationship is difficult to specify a priori (i.e., exactly how long is “longer” or “too long”) and likely varies depending on other task features, such as cue type, response modality, and task type.

Regarding *cue type*, pre-cue benefits have been observed with semantic (Bugg & Smallwood, 2016; Nuño and Bugg, 2026) and symbolic (Goldfarb & Henik, 2013; Jiménez et al., 2021) pre-cues. No study has directly contrasted these two cue types but it stands to reason that pre-cue benefits are most likely to be observed when pre-cues are semantic, thereby reducing if not eliminating demands (time and effort) on cue translation processes. Within the symbolic category, the findings of Jiménez et al. (2021) suggest that previous trial pre-cues that not only place demands on cue translation but also require cue translation processes to occur concurrent with stimulus processing on the preceding trial (as opposed to independent of it, such as during an intervening period) are highly unlikely to yield pre-cue benefits and should be avoided in future pre-cueing studies. To maximize the likelihood of observing pre-cue benefits, use of semantic pre-cues is recommended, or alternatively symbolic pre-cues paired with extra preparation time. One advantage of symbolic pre-cues, albeit one that no study to date has implemented, is that each pre-cue can serve equally often in each condition. For example, if a square or triangle is used to signal congruency, then researchers can counterbalance across participants which cue is linked to which trial type (congruent or incongruent trials), such that potential differences in cueing effects cannot be attributed to differences in the ease of pre-cue identification or translation, for example, between different symbols.

*Response modality* also appears to be important and may interact with cue type. In all studies except one that observed pre-cue benefits on incongruent trials (Bugg & Smallwood, 2016, Experiments 1 and 4; Jiménez et al., 2021, Experiment 7; Nuño and Bugg, 2026) or in mostly incongruent lists (though limited to the first trial; Bugg et al., 2015; Nuño et al., 2025), participants responded vocally and not manually (see Goldfarb & Henik, 2013, Experiment 2 for the exception) within a Stroop task. It may be harder to find pre-cue benefits in manual tasks because of the additional load associated with maintaining the arbitrary response mappings on top of the pre-cueing rules (i.e., which pre-cue is predictive of which trial type; Jiménez et al., 2021), especially when symbolic pre-cues are used. In addition to the working memory load associated with maintaining arbitrary S-R mappings in the manual Stroop task, the arbitrariness of these mappings also arguably alters the nature of the conflict that is present in the manual compared with the vocal task. While the vocal response (producing a spoken color) directly overlaps with both the relevant and irrelevant stimulus features, this source of conflict is not present with arbitrary button presses (Kornblum et al., 1990; Zhang et al., 1999). However, when Huang and Egner (in press) employed a manual (spatial) Stroop task with nonarbitrary responses, they nevertheless did not observe any pre-cue benefits, suggesting that the arbitrariness of response mappings is unlikely to be the sole driver of the fact that pre-cue benefits are more reliably observed in the vocal than in the manual Stroop task.

Another possibility is that it is not the use of a manual task with arbitrary S-R mappings that limited evidence of pre-cue use in prior experiments (Jiménez et al., 2021) but provision of a relatively small amount of preparation time. Indeed, Goldfarb and Henik (2013) found a pre-cue benefit on incongruent trials in a manual task with symbolic pre-cues, but participants had 1,500 to 2,500 ms to prepare, whereas in Jiménez et al. (2021), where there was no evidence of a pre-cue benefit with manual tasks (including those that used symbolic pre-cues), participants had only 750 ms. It may not be a single factor, but rather the combination of the additional load associated with maintenance of S-R mappings within a manual task and insufficient preparation time that is disadvantageous for observing pre-cue benefits (cf. Nuño & Bugg, 2026, for evidence of pre-cue benefits in a vocal task at a shorter CSI [1,100] when monetary incentives were available in the experiment).

The extant evidence also points to *task type* as another relevant task (external) factor given that evidence for pre-cue benefits, in trial- and list-level paradigms, has exclusively been observed in the color-word Stroop task (trial: Bugg & Smallwood, 2016, Experiments 1 and 4; Goldfarb & Henik, Experiment 2, 2013; Jiménez et al., 2021, Experiment 7; Nuño & Bugg, 2026; list: Bugg et al., 2015; Nuño et al., 2025). However, further research is needed to examine the validity of this claim since the bulk of published research on three- and four-choice tasks using the trial-level paradigm has used the Stroop task, and all studies but one using the list-level paradigm have used the Stroop task. There is only one published, trial-level study (albeit with three experiments) that used the Simon task (Wühr & Kunde, 2008), one trial-level study that used a spatial Stroop task (Huang & Egner, in press), and an unpublished study using trial- and list-level pre-cueing paradigms in the flanker task (Nuño & Bugg, 2025), none of which found pre-cue benefits. This was true despite their using 100% valid, semantic pre-cues and what appears to be ample preparation time based on prior research (e.g., Bugg & Smallwood, 2016). The findings from these experiments raise the possibility that it may be especially difficult for participants to engage a distractor-attenuating strategy to prevent being distracted by location in the Simon and spatial Stroop tasks or by the flankers in the flanker task, compared with attenuating processing of an irrelevant word in Stroop. Future research is critical for determining whether this conclusion is valid, or alternatively, whether pre-cue benefits on incongruent trials may be observed under select, yet unexplored, conditions in the Simon, spatial Stroop, and flanker tasks.

### Person (internal) factors

There are additionally two person (internal) factors that we believe to be influential in dictating pre-cue benefits. The person-related factors are variables that are internal to the participant, specifically motivation and ability. Identification of these factors was guided by synthesizing findings from our literature review and one additional observation. Our synthesis highlighted that pre-cue benefits on incongruent trials in two-choice task designs are reliably observed, as are benefits on congruent trials regardless of task design (but see Jiménez et al., 2021, 2024, for exceptions using previous trial pre-cues). In addition, as alluded to earlier, a key observation from the broader control literature is that there are highly reliable behavioral signatures of control adjustments observed in the same conflict tasks used in pre-cueing experiments, including congruency sequence effects and proportion congruence effects. A critical difference is that uniquely in the case of pre-cueing manipulations, participants must willfully engage distractor-attenuating mechanisms in response to an explicit pre-cue. By contrast, congruency sequence and proportion congruence effects are experience-driven (e.g., based on experiences with conflict; Botvinick et al., 2001) and likely operate implicitly outside of conscious awareness (Blais et al., 2012; King et al., 2012; van Gaal et al., 2010; see also Diede and Bugg, 2017). We posit that pre-cue benefits on incongruent trials in designs with more than two choices may uniquely depend on the outcome of a decision-making process, and that decision regards the question of whether the effort needed to willfully engage costly control processes in response to pre-cues is justified by the potential benefits of doing so, that is, whether participants are sufficiently motivated.

*Motivation* refers to whether participants are willing to expend effort to apply a cognitive strategy in response to the pre-cue (see, e.g., Goldfarb & Henik, 2013). Ample evidence suggests that people avoid cognitive effort and consider the costs of cognitive processes alongside putative benefits (e.g., being able to respond more quickly when pre-cued) when making decisions about whether to engage costly control processes (e.g., Kool et al., 2010; Shenhav et al., 2013). Here, it is important to recognize that participants can typically complete the tasks used in pre-cueing paradigms adequately without using pre-cues, making cue utilization cognitively demanding while offering potentially minimal perceived additional value. Incentives (e.g., rewards for high performance) may therefore be important in revealing pre-cue benefits, and we posit that this is especially the case in three- and four-choice tasks on the reasonable assumption that engagement of a distractor-attenuating strategy is more effortful than a distractor-enhancing strategy, given the latter involves attending to the dimension that is processed more automatically (i.e., participants readily use a distractor-enhancing strategy and the robustness of pre-cue benefits in two-choice tasks is consistent with that assumption). Few studies have examined the role of motivation in pre-cue benefits in three- and four-choice tasks but there is preliminary evidence that motivation matters (see Nuño and Bugg, 2026, for trial-level pre-cueing findings; see Bugg et al., 2015, Experiment 4; Nuño et al., 2025, for list-level pre-cueing findings, albeit findings that are restricted in showing a benefit for the first trial only), and merits systematic investigation in future studies.

One might question the putative role of motivation, however, given that some trial-level pre-cueing studies have demonstrated pre-cue benefits on incongruent trials without incentivizing good performance (Bugg & Smallwood, 2016; Goldfarb & Henik, 2013; Jiménez et al., 2021). While this is true, it does not negate two possibilities. One is that pre-cue benefits overall might be more robust or stable if participants were motivated to use the pre-cues. That is, some of the mixed evidence for pre-cue benefits across studies may reflect variation in participants’ motivation overall or fluctuations during the experiment.

The second possibility is that incentives may produce pre-cue benefits under some conditions in which such benefits are not otherwise observed. Suggestive of such a possibility, Nuño and Bugg (2026) found evidence for pre-cue benefits on incongruent trials at a 1,100 ms CSI when a monetary reward was available or participants were aware of the potential for a monetary reward; in contrast, when no rewards were available/there was no knowledge of a potential reward, Bugg and Smallwood (2016) did not find evidence for a pre-cue benefit on incongruent trials at the same or a similar CSI (but see Footnote 9). Another way in which motivation might interact with task (external) factors is in producing benefits in conditions that are particularly taxing, such as when a manual task requires the additional effort of maintaining the arbitrary S-R mappings. Furthermore, motivational incentives may be especially important in the list-level paradigm (Bugg et al., 2015; Nuño et al., 2025), where the congruency of each trial is only probabilistically predicted by the pre-cue and a distractor-attenuating strategy must be sustained across trials.

Tests of these possibilities in future studies should be mindful of the differential effects of reward on cognitive control, depending on how researchers implement incentive manipulations (Fröber & Dreisbach, 2014; Notebaert & Braem, 2015). Our conceptualization of the role that rewards may play in enhancing motivation in pre-cueing paradigms maps onto use of a performance-contingent reward prospect. In other words, the prospect of potentially earning a reward on a subset of trials or within certain blocks should motivate participants to perform at their best, including expending effort to use the pre-cues in a proactive manner (see Fröber & Dreisbach, 2014, 2016, for evidence that performance-noncontingent rewards can in contrast, reduce proactive control). When performance-contingent rewards instead are delivered as feedback (rather than a prospect), they act as a form of reinforcement learning. For example, if a reward follows a fast and accurate response, then participants may try to exploit the associations that led to the reward on later trials. While such rewards affect cognitive control (e.g., increasing the congruency sequence effect; Braem et al., 2012) and in theory may affect performance in a pre-cueing paradigm (e.g., if a participant is provided with feedback in the form of reward when performance is high on cued incongruent trials, this might strengthen associations between the pre-cue and distractor-attenuating strategies that were presumably used when responding), it is the more future-oriented prospect of reward that we expect to enhance pre-cue use.

According to TEPID, the second critical person (internal) factor is *ability*, which refers to whether participants can translate the pre-cue (infer what it means), identify a cognitive strategy that is appropriate for an incongruent trial or mostly incongruent list under conditions of stimulus uncertainty, and apply that strategy. Evidence of pre-cue benefits in three- and four-choice tasks suggests people are able to do this, at least transiently and under select conditions; however, ability, like motivation, may be a constraint that is contributing to (1) the overall limited evidence for trial-level pre-cue benefits (e.g., within an experiment people vary in their ability to construct and engage a distractor-attenuating strategy) and (2) the lack of any evidence for list-level pre-cueing benefits, which may point to an inability to sustain strategy use across trials. While a lack of ability may reflect difficulty translating the pre-cue specifically in the case of previous trial pre-cues (due to concurrent demands), we think differences in ability are more likely to manifest in two other ways.

The first way is that participants may have difficulty identifying an appropriate cognitive strategy—that is, a layperson participating in a pre-cueing study may not be privy to distractor-attenuating strategies. In contrast, distractor-enhancing strategies that reliably produce benefits on congruent and incongruent trials in two-choice tasks (reading and response prediction, respectively) may be more obvious and/or easily adopted. To remedy this, researchers might provide more thorough instructions regarding how to use each of the pre-cues, thereby alleviating the need for participants to identify such processes on their own.

Of note, Desender (2018), albeit using a two-choice task, demonstrated that instructing participants to use different strategies in response to list-level pre-cues led to expectedly different performance patterns. Participants performed five lists of a Simon task, each of which was 50% congruent and comprised 80 trials. They were asked to implement one of four distinct strategies at the beginning of each list, and performance was compared with a baseline list in which no strategy was assigned. There was a liberal strategy (respond as quickly as possible and trust intuition), a conservative strategy (respond as carefully as possible), a mostly congruent strategy (informed that most of the time the color patch would match the hand response and told to use that information proactively), a mostly incongruent strategy (informed that most of the time the color patch would not match the hand response and told to use that information proactively). Response time results revealed that congruency effects only differed from baseline in the mostly incongruent strategy condition, with a reduced congruency effect associated with the strategy. Error rate results indicated fewer errors for the conservative strategy, more errors in the liberal and mostly congruent strategies, and more errors on congruent than incongruent trials in the mostly incongruent strategy, relative to baseline (Desender, 2018). The findings regarding the mostly incongruent strategy lend initial support to the prediction that enhancing ability through instructions may lead to pre-cue benefits in mostly incongruent lists.

The second way that differences in ability may manifest is that participants may know what to do (e.g., try to filter out the distracting word in Stroop or ignore the location of a stimulus in Simon) but may not be able to do it. A reader might object, given the evidence in the broader cognitive control literature described above, pointing to reliable signatures of adjustments in control based on experience. However, willfully enacting an attentional bias may be fundamentally different than the application of an attentional bias that is shaped based on experiences during a task (e.g., encountering distraction frequently). Future research might reconceptualize the research question to ask, “Can we train people to use pre-cues more effectively?” When conceptualized in this way, the experimental design takes a different form where participants initially take part in a training phase (except for in a control group) before performing a test phase of the pre-cueing paradigm to determine if there is more robust evidence for pre-cue benefits following training. Researchers might vary the type of approach adopted in this training phase, comparing an unguided condition with a guided condition where factors such as feedback and the nature of instructions could be manipulated. Such experiments would provide insights into the role of ability and the question of whether pre-cue use is trainable, which has important applied implications.

These person (internal) factors likely interact with each other, and task (external) factors, as illustrated through examples related to motivation above. For example, if participants were unable to use pre-cues (e.g., they cannot apply a distractor-attenuating strategy), then providing an incentive to motivate pre-cue use would be unlikely to yield a benefit (see next section for additional examples of interactivity).

### A two-phase model of pre-cue use capturing the interactivity of task (external) and person (internal) factors

We posit that the task-related (external) and person-related (internal) factors of the TEPID framework may operate in two phases as illustrated in Fig. 4: preparation and strategy implementation. The first phase—preparation—begins with presentation of the pre-cue, and involves cue translation, identifying and preparing an appropriate strategy (akin to formation or reinforcement of a task set), and a metacognitive assessment of motivation and ability. During cue translation, participants encode the pre-cue and transform it into their expectancy regarding the congruency status of the upcoming stimulus. This process is affected by cue type and instruction clarity. A symbolic pre-cue (especially previous trial pre-cues) or vague instructions may hinder effective cue translation. Successful cue translation may lead to a valid expectation about what is to come, which guides the identification of an appropriate strategy and preparation of the strategy. In the three- and four-choice designs where incongruent trials (or mostly incongruent lists) are cued, this corresponds to identifying and preparing a distractor-attenuating strategy (i.e., down-weighting the task-irrelevant dimension and/or amplifying attention to the task-relevant dimension).

Critically, we propose that participants also perform a *metacognitive assessment* (a motivation and ability check) during the preparation phase. Here, metacognition refers to how people use judgments about their motivation/ability, and/or awareness of the ways that task demands (e.g., preparation time) interact with motivation/ability to guide decisions about pre-cue use. Motivation wise, we assume participants weigh the benefits and costs of adjusting their cognitive control based on the pre-cue. The need to engage a more effortful strategy (such as distractor-attenuation) may discourage engagement with the pre-cues or abandonment of them. Conversely, incentives like the prospect of earning a monetary reward might motivate pre-cue use.

Ability wise, judgments about two types of abilities are relevant. One is general ability to perform the task at hand (e.g., being able to quickly name colors in Stroop or respond to the central target in flanker). If a participant believes they are very good at the task or the task is easy enough without the pre-cues (e.g., during the uncued condition), then they may think that using the pre-cues is unnecessary. Experimentally, researchers could attempt to influence metacognitive assessments of general ability by, for example, having participants perform a more difficult version of the conflict task (e.g., with a short response deadline) in a baseline phase, which would presumably promote the perception that cue use is required to perform adequately in the subsequent test phase. The second is ability to use the pre-cues. If a participant believes they cannot identify or prepare an effective strategy, then this would discourage pre-cue use. Note that in all pre-cueing studies reviewed herein, judgments about ability are entirely subjective (e.g., based on intuitions that exist pre-experimentally, or after some practice, or based on how well it feels like one is performing during the task with and without pre-cues) because no feedback is provided to participants about their performance. The only exceptions are studies that have used point-based (Bugg et al., 2015) or monetary incentives (Nuño & Bugg, 2026), where participants received feedback indicating whether they earned a reward or not (indicating they performed better than in a baseline condition). It is possible that pre-cue use was shaped in part by this feedback rather than a motivational mechanism per se, as the feedback may have helped participants learn to apply an appropriate strategy or enhance their belief in their ability to engage the strategy. In short, if during the metacognitive assessment, participants judge that they are not motivated or able to use the pre-cues, then an absence of pre-cue benefits would not be surprising.

The second phase—strategy implementation—assumes that participants have had sufficient time to prepare the chosen strategy and are motivated and able to do so. Insufficient preparation time might prevent successful implementation of a distractor-attenuating strategy, but not distractor-enhancing strategies (response prediction), which may be entirely reactive (see Logan & Zbrodoff, 1982, for benefits even at very short CSIs in two-choice tasks; see Bugg & Smallwood, 2016, Experiment 2, for benefits regardless of CSI when distractor-enhancing strategies could be used). However, even when there is sufficient time to prepare a distractor-attenuating strategy, preparation likely varies across trials due to fluctuations in motivation, for example. On some trials, there may be a “failure-to-engage” preparatory processes (de Jong, 2000), such that preparation and execution of the strategy would have to occur in tandem during the second phase (after stimulus onset). Pre-cue benefits would be less likely on such trials.

This two-phase model is logical and empirically grounded but has not yet been tested. One might ask whether the key factors identified by TEPID influence each phase separately. Some task (external) factors, by definition, are linked to the first phase, such as cue type and preparation time. However, response modality and task type may influence pre-cue use in either or both stages. For example, it may be especially challenging to find pre-cue benefits in tasks in which the irrelevant dimension strongly captures attention at the time of stimulus onset. That may correspond to an effect of task type on strategy execution in the second phase; however, participants may give up on preparing a strategy in tasks like this (corresponding to an effect of task type on the first phase). The person (internal) factors of motivation and ability are directly referenced in the first phase of the model, both in influencing strategy preparation and factoring into the metacognitive assessment, but these factors may also have downstream effects on strategy execution in the second phase. Again, thinking about attentional capture, perhaps part of the ability that contributes to effective pre-cue use is maintaining the distractor-attenuating strategy in the face of strong bottom-up capture by the distractor, which may depend on these person (internal) factors.

### Design limitations

Finally, TEPID acknowledges that limitations of typical pre-cueing designs (the trial-level and list-level pre-cueing paradigms) may also be contributing to the limited evidence for pre-cue benefits to date. One potential limitation regards the uncued (control) condition, in which an uninformative pre-cue is presented (e.g., the word ATTENTION or a string of *X*s) that does not predict the congruency or proportion congruency of the upcoming trial/list. Researchers assume that, because the cue is uninformative, participants will not use it to prepare and thus it represents an appropriate comparison (control) condition against which to evaluate pre-cue use in the cued condition. However, this assumption may be flawed. Uninformative pre-cues inherently signal unpredictability. Participants might heighten control in response to the pre-cue because of the uncertainty it evokes, which may indicate a need to engage control (Mushtaq et al., 2011). Participants may also be inclined to heighten control in response to an uninformative pre-cue for another reason—so as not to risk being unprepared when a more difficult incongruent trial occurs, which will happen 50% of the time.

The key point is the following: If participants heighten control in response to the pre-cues in the uncued condition, for whatever reason (e.g., uncertainty; greater experience-dependent heightening of control in the absence of pre-cues), then naturally this would weaken evidence for pre-cue use (i.e., the difference in performance between the cued and uncued condition would be minimized). Future research using methods that can capture preparatory processes engaged in response to informative and uninformative pre-cues such as pupillometry and electroencephalography (EEG) would be well suited to determine whether use of an alternative design is recommended (though, a solution is not obvious for the most common design in which pre-cues are 100% valid).

A second design limitation pertains to list (block) composition, or how frequently participants encounter congruent and incongruent trials in cued and uncued conditions. Much evidence has demonstrated that in lists with frequent conflict (i.e., mostly incongruent lists) control is heightened (relative to lists with infrequent conflict) based on experience (for review see Bugg & Crump, 2012). Accordingly, it has been posited that there may not be much room for an additional heightening of control in response to a pre-cue, and consistent with this view, Goldfarb and Henik (2013) only observed a trial-level pre-cue benefit on incongruent trials in a mostly neutral list (and not a mostly incongruent list or 50% neutral/incongruent list). This limitation may be unlikely to explain the limited evidence for pre-cue benefits in trial-level paradigms which are 50% congruent. In contrast, and critically, the minimal evidence for pre-cue benefits in mostly incongruent lists in the list-level paradigm may be influenced by this design limitation. Recall that the key comparison is between a cued mostly incongruent list and an uncued mostly incongruent list (i.e., one preceded by ?????). In both lists, experience with frequent incongruent trials, which typically occur 80% of the time, should in and of itself heighten control and reduce the congruency effect. If the congruency effect is consequently at or close to floor, then any heightening of control based on the knowledge that the list will be mostly incongruent (i.e., the pre-cue) may yield little observable benefit. An initial test of the redundancy hypothesis using the list-level paradigm did not support the view that use of mostly incongruent lists is a primary limitation for observing pre-cue benefits (Suh et al., 2022; see also Jiménez et al., 2024, for findings that challenge the hypothesis from a trial-level pre-cueing manipulation in mostly congruent and mostly incongruent lists), but at least some evidence suggests it merits further consideration (Nuño et al., 2025).

A third design limitation is that pre-cue benefits are examined in the context of paradigms that typically require participants to frequently update or switch their strategies and/or attentional biases. Consider the typical trial-level design in which pre-cues signaling congruent and incongruent trials are randomly intermixed or are encountered in the same block as the non-informative pre-cue that maps onto the uncued condition. Similarly, though less severe, consider the typical list-level design in which pre-cues signaling mostly congruent and mostly incongruent lists are randomly intermixed along with uninformative pre-cues signaling that the next list type is unpredictable. Let us assume participants commit to engaging with each pre-cue and thus modulate which strategy/attentional bias they employ based on the pre-cue presented on each trial or in advance of each list. This means participants are frequently updating which strategy/bias is active (and possibly inhibiting the alternative), as they switch trial-to-trial or list-by-list between a distractor-enhancing strategy (which may include reading in a Stroop task) and a distractor-attenuating strategy (and in some studies, also switching on occasion to the strategy participants adopt in the uncued condition). Considering that adapting control states itself may carry a cost (Grahek et al., 2025; see also Kukkonen et al., 2025, for evidence that adaptations to at least some cues [those signaling reward] within a conflict task are more likely to occur when the cue applies to multiple future trials rather than a single trial), and people are disinclined to put forth the effort to switch between attentional states (Ileri-Tayar et al., 2025), it may be easier to simply ignore the cues, in which case pre-cue benefits would not be expected. Designing around this limitation while using 100% valid pre-cues by blocking each pre-cue is not a viable option,^11^ but an approach that might minimize the effects of such switching (which we believe to be more problematic in trial-level than list-level designs) is to increase the response-to-stimulus interval. The contrasting findings of Experiments 7 and 10 in Jiménez et al., (2021) raised the intriguing possibility that preparation time is not the only trial-level timing element that may dictate pre-cue benefits. Having sufficient time to unload the strategy used on trial *n* – 1 or sufficient time for it to dissipate before receiving the pre-cue for the next trial may additionally be important, and merits systematic investigation in future research.

A final design limitation we wish to highlight also is of greater concern in trial-level paradigms, namely, the common use of a single cue in the uncued condition but multiple cues in the cued condition (one for congruent and another for incongruent trials, and sometimes a third for neutral trials). This factor is particularly salient when the cued and uncued conditions are presented in separate blocks. In the cued block, the randomly changing nature of the pre-cues across trials likely attracts some attention, potentially detracting from the time available to prepare and slowing performance. However, in the uncued block, participants encounter the same cue (e.g., ATTENTION) repeatedly such that no such capture of attention would occur. Participants might be using pre-cues to a greater degree than is evident from calculating the difference between the cued and uncued conditions, if speeding in the cued condition based on pre-cue use is negated in part by slowing associated with the frequently changing pre-cues. One might posit that the consistent evidence for pre-cue use on congruent trials seems to counter this claim; however, incongruent trials should be most negatively affected because they are more dependent on preparation time (i.e., distractor-attenuating strategies rely more on ample preparation than distractor-enhancing strategies, which may be entirely reactive; Bugg & Smallwood, 2016). A simple solution is to vary the pre-cues in the uncued condition (e.g., ATTENTION and READY), while ensuring they still do not predict congruency.

Note that TEPID acknowledges that some of the factors discussed above might reflect a combination of task (external), person (internal), and/or design features. For example, one could conceptualize studies that involve incentive manipulations as providing insights into the task (external) factor of incentive availability, or the person (internal) factor of motivation. Similarly, one could conceptualize differences in how frequently participants must switch between different strategies or attentional biases, due to variations in design (e.g., trial vs. list-level; blocked vs. random pre-cues), as providing insights into the person (internal) factor of effort avoidance or design limitations.

## Connections to related literatures

In this section of our review, we ask two questions: how is the puzzle of profitless pre-cues and the TEPID framework informed by other literatures (confirming or challenging our claims)? Are there ideas from other literatures about conditions under which people use advance information to adjust attention that could be incorporated in studies examining congruency pre-cues? We begin with literatures that provide advance information about concrete features of upcoming stimuli, before transitioning to literatures in which the pre-cues are more akin to congruency pre-cues, therefore providing more abstract information and requiring nonspecific attentional biasing processes to reap a pre-cue benefit.

### Distractor priming

The first iteration of the distractor priming paradigm was a primed Stroop task, which entails a unique form of pre-cueing in which a prime revealing the upcoming distractor word (e.g., GREEN in black^12^) is shown prior to the Stroop stimulus (e.g., GREEN in red). Unlike congruency pre-cues, the prime provides participants with advance knowledge of the distractor’s identity, in principle allowing them to prepare to ignore the specific word that will appear on the next trial. Early work by Dyer (1971) showed that increasing the interval between prime and stimulus (0–500 ms) reduced interference effects (incongruent – neutral), suggesting reduced susceptibility to the distracting word with more preparation time. Facilitation effects (congruent – neutral) were also observed but did not increase with preparation time. These findings suggest that the prime enables participants to engage concrete, word-specific preparation processes that aid in minimizing interference (see also Chao, 2011, for converging evidence), and these processes can be engaged relatively quickly compared with those that are needed to produce pre-cue benefits when congruency is cued in three- and four-choice tasks (i.e., when the distractor’s identity is not known).

Recently, Curtis et al. (2025) extended this research by comparing distractor-primed conditions to neutral-primed conditions (e.g., #####), whereas prior work used a 0-ms preparation interval (Dyer, 1971) or an invalid prime (i.e., color word that did not match the distractor word; Chao, 2011) as the standard comparison condition. Curtis et al. found that distractor priming fully eliminated the interference effect, suggesting participants were able to proactively disengage attention from the word when it was presented in advance. In the typical Stroop task (and in their neutral-primed condition) the word appears concurrent with the color (i.e., the Stroop stimulus is an integrated object) such that attention is also captured by the word, making it difficult to selectively attend to the color (La Heij et al., 2001; Lamers & Roelofs, 2007). According to Curtis et al., priming the distractor word in advance disrupts this integration. This account may provide insight into why use of a distractor-attenuating strategy in response to a congruency pre-cue in a three- or four-choice task may not yield pre-cue benefits—because participants do not know what feature (e.g., word in the Stroop task) to ignore, it may be difficult to prevent the capture of attention by the distractor when the Stroop stimulus appears. Others have posited that the reason for the reduced interference effect when priming the distractor is because of an active inhibition of the primed distractor (Chao, 2011). However, as Curtis et al. noted, active inhibition of the primed distractor should not be accompanied by a facilitation effect since the word is supposed to be actively ignored. Yet facilitation effects have been observed (Curtis et al., 2025; Dyer, 1971).

Distractor priming has also been investigated in the Simon task, where relative position (i.e., relative to fixation) and side (i.e., relative to the side of the screen) have served as primes (Umiltà & Liotti, 1987). When a prime signaled only the relative position or only the side the stimulus would appear, effects of the prime were observed for the corresponding dimension. That is, a complete absence of a Simon effect was selectively found in conditions in which the to-be-ignored dimension was primed (see Experiment 5 of Umiltà & Liotti, 1987). These results are consistent with the distractor priming findings from the Stroop task (Chao, 2011; Curtis et al., 2025; Dyer, 1971), suggesting that pre-cueing the specific irrelevant dimension in the form of a prime can eliminate or attenuate interference effects.

Together, studies on distractor priming in Stroop and Simon tasks (Chao, 2011; Curtis et al., 2025; Dyer, 1971; Umiltà & Liotti, 1987) demonstrate that pre-cueing specific distractors via a prime can reduce or eliminate interference. These effects appear to depend on participants being able to disengage from known distractor features prior to stimulus onset, which is impossible with congruency pre-cues. Therefore, these findings suggest that, at least in part, the limited pre-cueing benefits observed in congruency pre-cueing paradigms might be attributable to the challenge of having to separate the irrelevant (e.g., word in Stroop) and relevant (e.g., color in Stroop) dimensions (Curtis et al., 2025) or difficulties actively inhibiting an unspecified feature (Chao, 2011).

### Cueing of spatial attention

Pre-cues are also widely employed in visual attention research (e.g., Coull & Nobre, 1998; Giordano et al., 2009; Müller & Rabbitt 1989; Pestilli, et al., 2007). A classic example is the Posner cueing task, where a probabilistic pre-cue signals the most likely location of an upcoming target (Posner, 1980). Cue use is quantified by comparing performance in validly cued trials to that on invalidly or neutrally cued ones. Spatial cueing tasks traditionally involve one of two types of pre-cues, endogenous or exogenous. Endogenous pre-cues, such as a centrally presented arrow pointing left or right, are meant to engage top-down, volitional attention, requiring participants to interpret the cue and deliberately shift their attention to the cued location. In contrast, exogenous pre-cues, typically consisting of briefly flashed stimuli in one of the possible target locations, are thought to trigger automatic attention shifts, relying on reflexive, bottom-up processes beyond conscious control (for a detailed review, see Carrasco, 2011).

In exploring the efficacy of congruency pre-cues, endogenous spatial attention pre-cues are particularly relevant since both types of cues involve intentional adjustments to attentional control. Unlike congruency pre-cues, however, endogenous spatial pre-cues have consistently proven effective in orienting attention, resulting in faster and more accurate responses for valid cues compared with neutral ones (Chica et al., 2013; Fernández et al., 2022; Giordano et al., 2009; T. Liu et al., 2007; Theeuwes 1991). Why does endogenous cuing of target stimulus location result in highly reliable effects when cuing of stimulus congruency does not? This difference in effectiveness likely stems from differences in the type of information the pre-cues provide, and thus, what sort of cognitive operations their use requires. Spatial pre-cues are arguably more direct in that they entail information about a single, concrete feature of the forthcoming target stimulus (its location). This seems to allow participants to efficiently translate the cue information into a spatial shift of focal attention. In contrast, congruency pre-cues are more indirect or abstract in nature, in that they provide information about the relationship of two stimulus features (word-meaning and color information being incompatible with each other), without revealing the specifics of either feature (e.g., that the stimulus is the word RED printed in blue ink). Accordingly, the mapping from the cue to a corresponding attentional adjustment is less straightforward (and perhaps more effortful) in the case of congruency pre-cues. According to classic models of the Stroop task (e.g., Botvinick et al., 2001; Cohen et al., 1990), the use of such cues would play out at the level of biasing broad feature channels (upweighting the processing of color information and downweighing the processing of word dimension) rather than attentional tuning to a concrete feature (“blue”).

### Pre-cueing of task switches

Targeting a different component of cognitive control than conflict paradigms, task switching paradigms examine the ability to alternate between distinct task sets (Allport et al., 1994; Rogers & Monsell, 1995). Typically, participants perform two tasks on a shared set of stimuli (e.g., judging the parity or magnitude of numbers), with response times compared across repetition trials (e.g., Task A follows Task A) and switch trials (e.g., Task A follows Task B). A robust finding is that switch trials elicit slower responses—so-called *switch costs* (reviewed in Monsell, 2003).

Cued task switching paradigms often use task specific pre-cues (e.g., ODD/EVEN) to indicate the upcoming task, and these cues provide concrete information about which task and stimulus features will be relevant (and which will be irrelevant). Notably, longer preparation intervals reduce—though do not eliminate—switch costs (Koch, 2003; Meiran, 1996; see also Meiran et al., 2000; Sudevan & Taylor, 1987), with the residual switch cost suggesting some switch processes may operate only after the stimulus appears (Longman et al., 2014; Monsell et al., 2003; Rogers & Monsell, 1995). Still, the reduction in switch costs with longer preparatory intervals suggests a role for task-set reconfiguration processes occurring before the stimulus, an example of effective use of proactive control based on pre-cue information.^13^

Of particular interest to the present review is the question of whether a *transition pre-cue* signaling an upcoming switch trial benefits performance when the identity of the upcoming task is not specified by the pre-cue and otherwise cannot be inferred^14^, as this is a condition more closely comparable to congruency pre-cues. These conditions are met when more than two tasks are employed. Critically, participants cannot pre-activate a specific task set in this case but instead must adopt a general readiness to switch to gain a pre-cue benefit (e.g., by broadly activating task rules for the probable tasks to be switched to and/or downregulating the recently performed task), since the relevant task only becomes known upon stimulus onset (e.g., signaled by stimulus color). Early work demonstrated that transition pre-cues produce larger switch costs than task specific pre-cues (Dreisbach et al., 2002, Experiment 4; see also Nicholson et al., 2006). Later work contrasted cued and uncued conditions (i.e., where switch/repetition likelihood is unknown) finding that on switch trials, transition pre-cues did not provide an advantage compared with the uncued condition; however, pre-cues signaling the specific task to be performed did provide an advantage (Karayanidis et al., 2009). These patterns are consistent with the notion that pre-cues requiring a nonspecific heightening of control are not as effective as pre-cues that provide more concrete information about the upcoming trial (see Chang et al., 2020; Koch, 2003, for examples), similar to the distinction between the effectiveness of congruency pre-cues signaling an incongruent trial in four-choice and two-choice tasks, respectively.

In addition to studies examining trial-level pre-cues, some studies have also explored list-level pre-cues in task-switching paradigms, which signal the likelihood of switching within an upcoming list (block). For example, Dreisbach and Haider (2006) compared list-level and trial-level pre-cues indicating 75% switch (high switch) probability or 75% repetition (low switch) probability in lists that were mostly switch trials or mostly repetition trials, respectively (see also Liu & Yeung, 2020). When list-level pre-cues signaled a high probability of switching, switch costs were reduced compared with pre-cues signaling a low probability of switching. However, this was driven primarily by slower response times on unexpected repetition trials in high switch lists and not by robust changes on switch trials. When trial-level pre-cues signaled a high probability of a switch, switch costs were extinguished compared with pre-cues signaling a low probability of a switch, and this was driven by faster response times for switch trials.

More recently, Liu and Yeung (2020) compared switch costs between 50% switch lists that were either preceded by a high switch probability pre-cue or a low switch probability pre-cue. That is, the effects of pre-cues were examined unbiased by experience with switch frequency (i.e., lists were equated in actual switch frequency; cf. Bugg et al., 2015). Across several experiments, there was a nonsignificant difference in switch costs between the pre-cues in 50% switch lists, suggesting that the pre-cues were not used to modulate switch readiness in these lists. However, error rate analyses suggested reduced switch costs when pre-cues signaled a high switch probability compared with low switch probability. Critically, however, the designs of both studies (Dreisbach & Haider, 2006; Liu & Yeung, 2020) entailed two tasks, limiting conclusions about a general heightening of control in response to the pre-cues, and there was not an uncued condition in which participants experienced high and low switch lists but were not informed of list type or trial type for comparison.^15^

In sum, task switching studies parallel conflict paradigms in demonstrating that, in designs in which a pre-cue benefit depends on a nonspecific heightening of control (i.e., when there are more than two tasks), there is little to no evidence of a pre-cue-benefit for switching in trial-level (Dreisbach et al., 2002; Karayanidis et al., 2009; Nicholson et al., 2006) or list-level paradigms (Dreisbach & Haider, 2006; Liu & Yeung, 2020). Another parallel of note is that task switching is nevertheless subject to reliable experience-driven adjustments in switch-readiness in response to varying the proportion of switch trials across lists (a list-wide proportion switch effect; e.g., Siqi-Liu & Egner, 2020) in the absence of transition pre-cues (see also Xu et al., 2024, for evidence of a modulation of the switch cost based on learned environmental cues). However, few studies have examined benefits of pre-cues using designs with more than two tasks or included uncued conditions with noninformative pre-cues. Thus, while pre-cue benefits are at present scarce, more work is needed in both trial- and list-level task switching pre-cueing studies to provide a fuller characterization of whether pre-cues can be used to heighten control and decrease the costs of switching.

### Proactive suppression in visual search

In conflict tasks such as the Stroop task, incongruent trials typically involve competition between two cognitive processes: a more automatic process, triggered involuntarily by the irrelevant stimulus feature (e.g., reading the word), and a volitional process, which requires deliberate, effortful selection (e.g., identifying the ink color). For an incongruency pre-cue to be effective, one would expect it to enhance volitional control and/or suppress the automatic response, allowing for more efficient resolution of conflict.

A similar form of competition has been extensively studied in the visual search literature. Here, participants are often asked to search for a target defined by pre-specified features in the presence or absence of a visually salient distractor in the display. Target selection relies on top-down, goal-driven control, but the salient distractor can capture attention, triggering a bottom-up selection process that interferes with efficient target identification. The canonical finding in this literature is a substantial distractor cost—a slowing of target identification in the presence of a salient distractor stimulus (Theeuwes, 1992, 2010).

A central recent debate in visual search concerns whether participants can effectively counteract this type of attentional capture by proactively suppressing salient distractors through top-down control (Luck et al., 2021). Studies have examined this by providing pre-cues indicating whether a distractor will be present in the search display and found that mere prior knowledge of distractor presence did not reduce distractor cost, indicating that participants may not be able to suppress distractors in a proactive manner (e.g., Bogaerts et al., 2022, Experiment 3; Moher et al., 2011). In fact, even studies that provided trial-by-trial pre-cues about the specific distractor location found no evidence that participants can effectively use this information to suppress attentional capture (Heuer & Schubö, 2020; Wang & Theeuwes, 2018; Zhao et al., 2023).

In another close resemblance to the congruency pre-cue literature, recent visual search research suggests that distractor suppression can be achieved through experience-based learning rather than cue-based proactive control (Goschy et al., 2014; Huang et al., 2021, 2022; Sauter et al., 2018; Theeuwes et al., 2022; Wang & Theeuwes, 2018). Specifically, studies have shown that when a distractor’s features remain fixed across trials, the attentional cost of its presence diminishes over time (Cunningham & Egeth, 2016; Vatterott & Vecera, 2012) and can even be inverted (Gaspelin et al., 2015; Gaspelin & Luck, 2018) suggesting a learned suppression mechanism. Conversely, when the distractor features vary unpredictably from trial to trial, distractor costs persist. Further evidence for learning-based distractor suppression comes from studies demonstrating reduced distractor costs in blocks of trials where the frequency or specific location of distractors is high compared with when it is low (Bogaerts et al., 2022; Huang et al., 2023; Wang & Theeuwes, 2018; Won et al., 2019), conceptually replicating the list-wide proportion congruency effect in cognitive conflict tasks.

In summary, visual search studies of distractor suppression, whose setup shares important features with cognitive conflict tasks, have also failed to provide evidence for an effective use of pre-cues to engage top-down control. Instead, successful distractor suppression appears to be experience-driven, relying perhaps on statistical learning mechanisms rather than on-demand, volitional regulation of attention.

## Discussion

We provided the first systematic review of the congruency pre-cueing literature. Our aim was to address the question of why congruency pre-cues are often profitless, especially under conditions of greatest theoretical and practical interest—when distraction is anticipated but uncertain (i.e., the distractor’s identity is unknown)—such that nonspecific attentional biasing (e.g., distractor-attenuating strategy) is required to benefit from the pre-cue. These conditions map onto the real-world examples presented at the outset of our paper (e.g., classroom, driving). We reviewed the contrasting findings between the two-choice literature, where participants routinely harness pre-cues to perform better in the face of distraction (i.e., incongruent trials) and the three- and four-choice literatures, where there is limited evidence for pre-cue benefits at the trial-level and no evidence at the list-level (beyond two studies showing an effect for the first trial of the list). A critical distinction between these literatures is that in two-choice tasks, a pre-cue benefit on incongruent trials does not depend on use of a distractor-attenuating strategy; rather, the distractor-enhancing strategy of response prediction can benefit performance. Thus, at the heart of the puzzle emerges the question of whether, or under what conditions, participants can utilize distractor-attenuating strategies in a preparatory fashion when a pre-cue signals impending but unknown distraction.

In addition to drawing a contrast with the two-choice literature, we also pointed out the contrast between the weak evidence for pre-cue benefits in three- and four-choice task designs and the strong evidence for experience-based adjustments in control (e.g., congruency sequence effects, list-wide and item-specific proportion congruence effects) that are reliably observed in the same conflict tasks (Stroop, flanker, Simon) and attributable to distractor-attenuating mechanisms. A critical distinction in this case is between mechanisms that operate implicitly based on experience (e.g., statistical learning) and a mechanism that operates explicitly in a willful fashion, with the latter being unique to pre-cueing paradigms (see “Theoretical and Practical Implications” section below for elaboration). These two contrasting patterns of findings within the pre-cueing and broader cognitive control literature, alongside the systematic review of the three- and four-choice literature, led us to propose the TEPID account, a novel theoretical framework for understanding the puzzle of profitless pre-cues (i.e., why pre-cue use has been tepid). Synthesizing the literature, we proposed several key task-related (external) factors (e.g., preparation time; response modality) and person-related (internal) factors (motivation and ability) that we believe to be most influential in dictating pre-cue benefits (or the absence thereof) and a two-stage model of how they interact to influence pre-cue use. Furthermore, the TEPID framework highlighted limitations of current research designs that may be contributing to the limited evidence for pre-cue benefits, formulated recommendations for future research to maximize evidence for pre-cue benefits, and proposed novel, testable predictions. For example, there has been little systematic inquiry into the interactivity of task-related factors (for an exception, see Jiménez et al., 2021) and little to no investigation of the role of person-related factors (motivation and ability) in pre-cue use. Further, few studies have tested the interactivity of task- and/or person-related factors, interactions that TEPID suggests to be critical in advancing understanding of pre-cue use.

A brief survey of related literatures reinforced conclusions that emerged from our review of the pre-cueing literature. For example, research utilizing distractor priming paradigms and spatial cueing paradigms both consistently found benefits of advance information. What these paradigms have in common is that the prime or pre-cue, respectively, conveys a concrete distractor or target feature of the upcoming stimulus (e.g., distractor word in Stroop priming paradigm or location of the stimulus in spatial pre-cueing paradigms). Accordingly, participants can efficiently translate the cue into an effective strategy, disintegration of the Stroop stimulus (Curtis et al., 2025) and/or active inhibition of the specific word indicated by the prime (Chao et al., 2011) in the Stroop priming paradigm, or a simple spatial shift of attention in the spatial cueing paradigm. In contrast, research examining pre-cueing of task switches and proactive suppression in visual search has found little or no evidence, respectively, for pre-cue benefits (enhanced switching, or a reduction in the costs of a visual distractor). The findings from these related literatures suggest that the puzzle of profitless pre-cues is not specific to the congruency pre-cueing literature. To the extent that the challenges faced by participants when preparing a nonspecific switch in a three-choice, pre-cued task switching paradigm or preparing to avoid an unknown visual distractor in a visual search paradigm are similar to the challenge of preparing a distractor-attenuating strategy in three- and four-choice congruency pre-cueing paradigms, TEPID has the potential to also guide future research in these related literatures. As in the congruency pre-cueing literature, there are relatively few studies in some areas (e.g., pre-cueing of task switches) and limited systematic investigation of the role of factors highlighted within the TEPID framework, including preparation time, motivation and ability.

We conclude this review first by discussing the theoretical and practical implications of the current evidence for pre-cue benefits. Then we expand upon the future research directions offered earlier (in section “TEPID: A Novel Framework for Understanding the Puzzle of Profitless Pre-Cues”) by considering broader recommendations for future research, including alternative methodologies researchers might bring to bear on the puzzle, some of which provide additional tests of predictions formulated by the TEPID framework.

### Theoretical and practical implications

Theoretically speaking, it has long been assumed that people can willfully bias attention in a goal-directed manner (e.g., Ach, 1910; Norman & Shallice, 1986; Posner & Snyder, 1975; Shiffrin & Schneider, 1977; but see Hommel, 2013, for an alternative view about the role of consciousness in control). More recently, the DMC account (Braver, 2012; Braver et al., 2007) put forth the idea that there are two control mechanisms, one of which is proactive and enables people to minimize distraction preemptively. One way in which people are thought to do this is by harnessing reliable cues that allow them to anticipate the occurrence of distraction and engaging preparatory attentional biasing mechanisms (i.e., before stimulus onset) to minimize detrimental effects of the distraction. Although more research is needed to determine whether conditions can be identified that more reliably lead to pre-cue benefits in three- and four-choice tasks, the limited evidence for pre-cue benefits in the trial-level paradigm paired with the lack of evidence for a list-level pre-cue benefit (but see Suh & Bugg, 2021, for evidence suggesting benefits may extend beyond a single trial under select conditions) cast serious doubt on the notion of an explicit proactive control mechanism that operates in a preparatory and/or sustained fashion, challenging the DMC account (Braver, 2012; Braver et al., 2007; De Pisapia & Braver, 2006). One possibility is that the willful control of attention in a proactive fashion is limited to conditions in which participants can harness concrete information provided in advance of a stimulus to enhance performance (e.g., distractor priming; spatial pre-cueing) or potentially prepare a distractor-*enhancing* strategy (e.g., to relax attention when expecting most trials in a list to be congruent), but these explicit proactive control processes are not able to operate in a nonspecific way (such as when the distractor identity is unknown; i.e., three- and four-choice tasks in congruency pre-cueing paradigms; pre-cued switching in a three-choice task paradigm; proactive suppression in visual search). The fact that there is some evidence for pre-cue benefits in three- and four-choice tasks that can be attributed to nonspecific distractor-attenuating strategies (trial-level: Bugg & Smallwood, 2016, Experiments 1 and 4; Goldfarb & Henik, 2013, Experiment 2; Jiménez et al., 2021, Experiment 7; Nuño and Bugg, 2026; list-level first trial: Bugg et al., 2015; Nuño et al., 2025) suggests this possibility may be unlikely. Instead, pre-cue benefits may depend on the presence of several key factors, as described in the TEPID framework, and the findings from studies testing the predictions of TEPID will lead to a more complete understanding of the willful biasing of attention and proactive control.

Not mutually exclusive is the possibility that pre-cue use may be a last resort, and not a default strategy people use in conflict paradigms. Bugg (2014) put forth the associations as antagonists to cognitive control (AATC) account to explain mixed evidence for the role of top-down processes in list-wide proportion congruence effects. She suggested that the use of top-down control is a last resort, and participants will instead harness more basic mechanisms (e.g., simple S-R learning) when they are available that can also lead to high levels of performance on the more difficult, incongruent trials. Applying AATC to the present puzzle, a factor that may be limiting evidence for pre-cue use is the fact that conflict paradigms enable adjustments in control outside of pre-cue use. For example, the mere influence of experiencing incongruent trials relatively frequently (even in a 50% congruent list, which most trial-level paradigms use) can lead to a heightening of control and relatively good performance. Consistent with AATC, participants may rely on these less effortful mechanisms (Bustos et al., 2024; Ileri-Tayar et al., 2025) and therefore not use the pre-cues to engage proactive control in an explicit fashion.

Consideration of these possibilities begs the question of why there is a general lack of evidence demonstrating pre-cue driven adjustments in control, while in the same conflict tasks there is robust evidence for experience-driven adjustments in control. For example, there is little evidence for trial-level pre-cue benefits and no evidence that participants can harness previous trial pre-cues to benefit performance, yet people reliably show reduced congruency effects on trials following an incongruent trial (the congruency sequence effect, reviewed in Egner, 2017). As another example, there is no evidence for a list-level pre-cue benefit, yet people reliably show reduced congruency effects in lists (blocks) in which incongruent trials are frequent compared with when they are rare (the list-wide proportion congruence effect, reviewed in Bugg & Crump, 2012; Egner, 2023) including in the list-level pre-cueing paradigm. These experience-driven performance signatures are supported by distractor-attenuating mechanisms (i.e., down weighting of the task-irrelevant dimension and/or amplification of task-relevant dimension, see Braem et al., 2019), yet such mechanisms are rarely evidenced in tasks using explicit congruency pre-cues (three- and four-choice task designs).

We posit that a critical difference is that uniquely in the case of pre-cueing manipulations, participants are tasked with willfully engaging distractor-attenuating mechanisms in response to an explicit pre-cue. By contrast, the congruency sequence and proportion congruence effects do not require explicit cue translation and likely operate implicitly (Blais et al., 2012; King et al., 2012; van Gaal et al., 2010; see also Bugg, 2017) based on associative learning (e.g., Hommel, 2013) and/or reinforcement learning mechanisms (e.g., Abrahamse et al., 2016; Botvinick et al., 2001; Egner, 2014; Verguts & Notebaert, 2009). For example, frequently experiencing incongruent trials within a list may shape a distractor-attenuating mechanism that is optimally tuned to the regularities of the list, such that the degree of down-weighting of the distractor dimension, for example, aligns with the learned likelihood of encountering conflict from that dimension. In contrast, when an incongruent trial or mostly incongruent list is pre-cued, it falls on the shoulders of the participant to prepare a distractor-attenuating strategy and determine how to down-weight the distractor, without support from learning processes. Some researchers have posited that control must be learned (rooted in experience) to observe flexible adaptations in parameters such as down-weighting irrelevant features (Braem et al., 2024). Others have suggested there is not a causal role for consciousness in control processes such as focusing attention after conflict (akin to processes underlying the congruency sequence effect and list-wide proportion congruence effect; Hommel, 2013; see also Kunde et al., 2012, for examples of putative instances of explicit control under conditions in which triggering stimuli are not consciously perceptible). Furthermore, some theories have posited that attempts to control automated procedures (akin to learning-based control parameters in conflict tasks) by shifting attention inward to consciously process the explicit knowledge of how the procedures work can harm performance (Masters & Maxwell, 2008). On these views, the limited evidence for pre-cue benefits may not be surprising.

Related to this critical difference, another explanation for the contrasting evidence when considering pre-cue driven adjustments in control and experience-driven adjustments, is motivation. As highlighted in the TEPID framework, pre-cue use may be uniquely dependent on the outcome of a decision-making process, as participants must decide whether to engage the effort needed to willfully prepare a distractor-attenuating strategy in response to a pre-cue. In contrast, experience-driven mechanisms are shaped by learning, potentially removing this decision point. The weaker evidence for pre-cue benefits may in part reflect participants deciding that the potential benefits are not worth the effort needed to effectively use a pre-cue.

Practically speaking, the limited evidence for pre-cue benefits raises doubts about the effectiveness of the various warnings we presented as real-world examples at the outset (e.g., a teacher’s imperative to pay attention; a road sign warning drivers not to get distracted) given that these examples entail expected but unspecified distraction(s). Especially doubtful is people’s ability to harness these warnings when distraction is likely over extended periods (e.g., high-traffic scenarios) given the lack of evidence for list-level pre-cue benefits. Here too, future research will be critical for determining the conditions under which such warnings may benefit performance. For example, it may be that highly motivated students or drivers would be apt to heed such warnings and prepare attention accordingly. At present, though, the ways in which these warnings are delivered in the real world do not appear to consider moderating factors like motivation. Researchers that are serious about informing real-world applications should consider what an optimal design is for investigating the question in the laboratory. There are at least three features of typical pre-cueing designs that do not map well onto real-world scenarios. One is that in the real world, distraction may be more likely to be probabilistic rather than certain. The second is that the occurrence of a distractor is almost certainly temporally less predictable in real-world settings than in current laboratory experiments, where trial-level timings permit participants to predict exactly when the distractor will appear (for an exception, see Bugg & Smallwood, 2016, Experiment 4, where CSI varied). The third is that, in real-world settings, when responding to a warning, such as from an automated driving system, one may be most inclined to slow down (be more cautious), yet the approach in the laboratory is to look for a speeding of performance as evidence of pre-cue use. Thus, future studies interested in translating evidence to the real-world should consider partially valid pre-cues, unpredictable distractor onsets, and alternative measures of pre-cue use that can index response caution (e.g., Drift Diffusion Modeling).

### Broader recommendations for future research

We encourage researchers to test predictions inspired by TEPID including by applying new methodologies to the study of pre-cue benefits. We briefly detail three such methodologies including reasons they may be fruitful in helping to solve the puzzle of profitless pre-cues but before we do so, we wish to address power as a potential explanatory factor in this puzzle.

A quick glance at Table 2 suggests that the sample sizes employed in some three- and four-choice studies appear to be relatively small, which may raise the concern that low statistical power contributed to the emergence of the puzzle at hand. However, all these studies employed within-subjects manipulations of cueing, which enhances power. Additionally, in all cases where we harnessed empirical findings to identify the main task-related factors at play in the pre-cueing puzzle (preparation time, response modality, task type), the studies revealed consistent patterns of pre-cue benefits or their absence across two (preparation time; Bugg & Smallwood, 2016) or more (use of manual/arbitrary responses; Huang & Egner, in press; Jiménez et al., 2021; use of Simon task; Wühr & Kunde, 2008) experiments, with two exceptions. One exception is the finding of Jiménez et al. (2021) of a pre-cue benefit using vocal responses, which as previously noted was not reproduced in another experiment in their study (though, in this case, there are cross-study replications of pre-cue benefits in vocal experiments from Bugg & Smallwood, 2016). The second exception is the finding of a pre-cue benefit using manual responses uniquely for incongruent trials in a mostly neutral list but not a mostly incongruent list (with list composition manipulated between subjects; Goldfarb & Henik, 2013). No study has reported an attempt to reproduce this finding, but our consideration of the role of list composition as a design limitation in the TEPID framework was additionally informed by other studies (Nuño et al., 2025; Suh et al., 2022). It is also important to note that the main factors we highlighted in TEPID were not solely inspired by findings from pre-cueing studies but have roots in broader findings from the control literature. For example, the idea that additional preparation time should facilitate configuration of an attentional strategy is grounded in prior theorizing about temporal constraints on proactive control (e.g., Braver et al., 2007; Meiran, 1996). Similarly, the idea that motivation should affect pre-cue use is grounded in prior theorizing about cost-benefit analyses influencing decisions about when to use cognitive control (e.g., Kool et al., 2010; Shenhav et al., 2013). In short, while we think it is advisable for future research on pre-cueing to be mindful of power, the picture emerging from the pre-cueing literature does not appear to be dependent on underpowered studies.

One methodology that future research might apply is to consider alternative behavioral signatures of pre-cue use. For example, one might examine how pre-cues affect signatures of experience-driven adjustments in control. Along these lines, Hutchison et al. (2016) used trial-level pre-cues embedded within a list-wide proportion congruence paradigm (Experiment 1) and an item-specific proportion congruence paradigm (Experiment 2) in vocal, color-word Stroop tasks. List-wide and item-specific proportion congruence effects depend on participants learning that certain lists or items (stimuli) tend to be congruent or tend to be incongruent, which requires learning about the correlation between words and colors (whether they match or conflict; cf. Melara & Algom, 2003). These proportion congruence effects were compared between cued and uncued conditions in Experiment 1 where pre-cues were 100% valid or between EASY and HARD pre-cues in Experiment 2 where the pre-cues were 70% valid. In both experiments, evidence for the experience-driven effects was modulated by pre-cueing. In Experiment 1, there were reduced list-wide and item-specific proportion congruence effects in the cued compared with uncued condition. In Experiment 2, there was a reduced item-specific proportion congruence effect for HARD pre-cues compared with EASY pre-cues. Although designs like this one are more complex than the typical pre-cueing design, they may be valuable for two reasons. One is that the findings suggest that participants used the pre-cues, which challenges a disuse explanation that remains possible for a lack of pre-cue benefits in some studies. The second is that such designs have the potential to facilitate theoretical understanding of whether explicit proactive control reliably affects experience-driven adjustments in control. The findings of Hutchison et al. might suggest that adjustments based on implicit regularities are not implemented (or are interfered with; cf. Masters & Maxwell, 2008) when explicit information is available to guide control. Applying the trial-based GLM analysis that affords a finer-grained examination of interactions between pre-cue use and learning processes may be valuable for questions like this (Suh & Bugg, 2021).

The second methodology is to apply cognitive neuroscience tools to the study of pre-cue effects. A major challenge within the broad pre-cueing literature on three- and four-choice conflict tasks is that the behavioral approach studies have employed does not allow researchers to identify and study signatures of preparatory processes post pre-cue, prior to stimulus onset, which are central to TEPID and theories of proactive control more generally. Thus, researchers using the behavioral approach can only assume that preparation was ineffective when no pre-cue benefit is observed. Yet signatures of preparation are an important source of information in better understanding the conditions under which pre-cue benefits are and are not observed. For example, the absence of a pre-cue benefit may reflect a failed attempt to use the pre-cue (e.g., preparation was incomplete prior to the stimulus appearing) or no attempt at all (e.g., the metacognitive assessment led participants to decide not to use the pre-cues) and this information is critical for testing theoretical accounts.

Pupillometry or EEG are the types of tools that might be harnessed to shed light on this issue. Both have been used to index preparatory activity in conflict tasks (pupillometry: Unsworth & Miller, 2023; cf. Hershman & Henik, 2019, 2020; e.g., EEG: e.g., Bianco et al., 2021; Gajewski et al., 2020; van den Berg et al., 2014; West & Alain, 2000). As an example, considering that changes in pupil dilation have been used as an indicator of effort expenditure (e.g., Kahneman, 1973), researchers could compare pupillary responses between theoretically motivated conditions of interest in pre-cueing paradigms according to the TEPID framework: for example, between varying CSIs; in tasks that vary with respect to load associated with maintenance of S-R mappings; and when incentives are available versus when they are not to look at effects of motivation. Taking a step back, researchers could also assess changes in pupil dilation to confirm (or disconfirm) some assumptions of current pre-cueing designs, such as the greater preparatory demands in three- and four-choice tasks compared with two-choice tasks, and the assumption that the uncued condition is truly a baseline in which preparation does not approximate that of the cued condition on incongruent trials. Along these lines, using a Bugg and Smallwood (2016) like pre-cueing paradigm in a manual color-word Stroop task with a 3000 ms CSI (but unfortunately no uncued condition for comparison), Unsworth and Miller (2023) showed that the pupillary response was larger when incongruent trials were pre-cued than when congruent trials were pre-cued, with the pupillary response increasing during the period of preparation. Additionally, a larger ramping up of pupillary responses in a preparatory fashion was uniquely associated with response times on incongruent trials (see also Hood et al., 2022, for an application of pupillometry in a cued anti-saccade task). Future studies should contrast these patterns across cued and uncued conditions, though it should be noted that a downside of pupillometry is a relatively low temporal resolution and potential confounds from luminance changes in the visual stimulation. Neither of these issues affect EEG, which—when paired with sophisticated analysis approaches—can provide trial-level and millisecond-resolved information about stimulus and task representations (e.g., Kikumoto & Mayr, 2020)

The third methodology is to adopt an individual differences approach. One reason for doing so is that the group level (mean) analyses may be missing out on a subset of participants who are using the pre-cues very effectively (see Nuño et al., 2025). While it can be said that for any empirical phenomenon, some participants likely will show an effect while others will not, we think the former group is theoretically important in the case of the pre-cueing puzzle because one conclusion that might be drawn based on the preponderance of evidence is that people simply cannot willfully use proactive control. However, if individual differences studies reveal that some people reliably produce pre-cue benefits, then the conclusion would be more nuanced. Rather than dismissing the possibility of willful proactive control, such patterns would suggest select people effectively engage proactive control in this manner.

A second reason for doing so is that an individual differences approach has the potential to identify moderators of pre-cue benefits. This presents an opportunity to test TEPID, which assumes that factors like motivation and ability serve as potential gatekeepers, such that those who are “high” in these factors should produce larger pre-cue benefits than those who are “low.” In addition to examining factors identified by TEPID, an obvious target is working memory capacity given longstanding evidence for its role in cognitive control including conflict tasks like Stroop (e.g., Kane & Engle, 2003). Interestingly, in Unsworth and Miller’s (2023) study, only individuals with high WMC demonstrated a larger pupillary response for cued incongruent compared with cued congruent trials during the preparatory interval. To the extent that the pupillary response is indicative of pre-cue use, this raises the possibility that a subset of participants did not use the pre-cues. If disuse of the pre-cues is prevalent more generally in pre-cueing experiments (assuming a decent-sized subset of low WMC individuals in each sample), it would perhaps be less surprising that the evidence for pre-cue benefits is so limited (but see Hood & Hutchison, 2021; Hood et al., 2022; Welhaf et al., 2025, for evidence that those *lower* in WMC are more likely to use a different type of hint called a goal-reminder, distributed periodically throughout a color-word Stroop task). The identification of moderating factors would also have practical significance. For example, it would be valuable to identify the characteristics of individuals who can reliably adjust attention on demand in military settings where it is essential to heighten control in response to an automated message or imperative from a superior.

### Conclusion

A systematic review of the congruency pre-cueing literature in conflict tasks revealed a puzzling lack of reliable benefits of 100% valid congruency pre-cues on the processing of incongruent stimuli, especially under circumstances where the exact features of the upcoming stimulus are unknown, and anticipatory attentional modulation would have to take the form of a dimensional attenuation of distractor-processing or amplification of target-processing. This set of findings casts serious doubts on common assumptions about people’s ability to volitionally engage proactive control and poses a challenge to some extant theories (e.g., DMC account). A synthesis of key findings considered alongside design parameters employed by the reviewed studies led us to propose a novel theoretical framework called TEPID. The framework incorporates a two-phase model that highlights the interactivity of several key task- and person-related factors that we argue determine the likelihood of successful pre-cue use. From this synthesis of relevant factors, we derived several design recommendations and testable predictions for future studies. Given that a similar lack of pre-cue benefits also characterizes related literatures on task switching and visual search, we hope that the TEPID framework can inform the interpretation and design of a wide range of investigations of proactive control using pre-cueing manipulations, and their translation to real-life applications (e.g., in classrooms, on highways).

## Data Availability

Not applicable (This is a theoretical/review article).

## Footnotes

1 Some studies also include neutral trials and calculate interference effects by comparing slowing in the incongruent condition to the neutral condition, as we’ll describe in more detail in the “Review of Literature” section.

2 In this review, for both the trial-level and list-level paradigms that we consider (see section “Pre-cueing Paradigms and Primary Outcomes of Interest”), we exclude studies that did not use 100% valid pre-cues. In the case of the trial-level paradigm, this resulted in exclusion of studies that used a probabilistic pre-cue (e.g., the next trial is 80% likely to be conflicting, see e.g., Gratton et al., 1992; Ghinescu et al., 2010) because on 20% of trials, the pre-cue did not accurately indicate the congruency of the next trial. In the list-level paradigm, we did not exclude studies that used probabilistic pre-cues so long as the pre-cues were 100% valid meaning that when the pre-cue said the next *list* would be 80% conflicting, it was 80% conflicting. In the list-level pre-cueing literature, most studies use precisely such cues because the goal is to compare a cued condition (that validly informs participants in advance that the list will be 80% conflicting) to an uncued condition (that does not provide this information but still entails an 80% conflicting list). However, some studies using list-level pre-cues employed partially valid (first half; Entel et al., 2014, Experiment 1) or invalid (Bugg et al., 2015, Experiment 5) pre-cues and these studies were excluded. None of the excluded studies reported evidence of pre-cue benefits for incongruent trials or mostly incongruent lists. These studies fall outside the scope of the present review because it is most puzzling not to find pre-cue benefits when cues are 100% valid.

3 See later subsection “Distractor Priming” within the section “Connections to Related Literatures” for a paradigm in which participants are told what the distractor will be on the next trial, thus affording use of a *specific* distractor-attenuating strategy.

4 Although we use this label for purposes of providing a clear contrast to the alternative strategy (distractor-enhancing), as noted above, the non-specific attentional mechanism we refer to as distractor-attenuating may involve down-weighting the task-irrelevant dimension and/or amplifying attention to the task-relevant dimension.

5 Distractor-enhancing strategies could also be used in the list-level pre-cueing paradigm regardless of task design. When a mostly congruent list is cued, participants could read the word on each trial that follows. However, they may not commit fully to word reading because incongruent trials occur occasionally following the pre-cue, and fully basing responses on distractor identity (i.e., reading) may produce high error rates on incongruent trials. Accordingly, the degree of distractor-enhancement (i.e., how much weight is given to the distractor dimension when responding) may not be as robust compared to when a congruent trial is expected (trial-level pre-cueing paradigm). When a mostly incongruent list is cued, the distractor-enhancing strategy of response prediction could be used to benefit performance if a two-choice design was used, but no studies employing the list-level pre-cueing paradigm have used a two-choice task.

6 In each experiment, half the participants received 100% valid pre-cues and half received partially (80%) valid pre-cues. Cue validity did not interact with any factors of interest and was not examined further in subsequent experiments (i.e., all other experiments in the study used 100% valid pre-cues).

7 The authors took inspiration from Bugg and Smallwood (2016), who demonstrated pre-cue benefits on incongruent as well as congruent trials, when designing this experiment. In Bugg and Smallwood’s vocal procedure, an experimenter coded what response participants vocalized, which added time between trials. This interval was intended to mimic that delay.

8 In Experiments 1 and 2, the relative frequency of the colors varied such that in the mostly congruent list, the colors predicting a subsequent congruent trial occurred more frequently and in the mostly incongruent list, the colors predicting a subsequent incongruent trial occurred more frequently. As the authors noted, this may lead both to a speed up after frequently occurring colors and a slow down after infrequent colors (i.e., after the colors predicting an incongruent/congruent trial in the mostly congruent/incongruent list, respectively). In these experiments, the cueing effects tended to be evident for the frequent but not infrequent colors, but this pattern may be attributed to this confound rather than effects of pre-cueing per se.

9 The contrasting findings between Bugg and Smallwood (2016) and Nuño and Bugg (2026) may provide additional support for the role of motivation in producing on-demand adjustments in control at an intermediate (1,100 ms) CSI considering Bugg and Smallwood did not observe pre-cue benefits at this CSI across two experiments in which incentives were not available. However, differences between these studies must be interpreted with caution because the sample sizes in Bugg and Smallwood were smaller than Nuño and Bugg, and therefore it is possible that lower power, rather than the absence of incentives, contributed to the lack of a pre-cue benefit at the 1,100 ms CSI in Bugg and Smallwood (although they consistently observed pre-cue benefits at a longer CSI).

10 Experiment 2 used the same design as Experiment 1 but additionally included 50% congruent lists, which were validly cued by a semantic pre-cue stating 50% MATCHING/CONFLICTING. As expected, there was no pre-cue benefit for these lists.

11 This would result in blocks that are 100% congruent or incongruent, eliminating the utility of the pre-cues.

12 Note that the color black is never part of the response set in these studies.

13 One might posit that pre-cueing congruency in conflict tasks is directly analogous to pre-cueing a task (with a task-specific pre-cue) in task-switching paradigms with bivalent stimuli, with each type of pre-cue informing participants about what dimension of the upcoming stimulus to attend to. A congruent pre-cue signals “Task 1” to respond with the help of the distractor and an incongruent pre-cue signals “Task 2” to respond by focusing solely on the target/ignoring the distractor, just like task-switching cues signal whether to focus on parity or magnitude of numbers, for example. Considering this analogy, it may be surprising that in conflict tasks, the pre-cues tend to produce no benefit at all, whereas in task-switching paradigms, there is a reduction in the switch cost but switching is incomplete such that a residual cost remains. One potential reason for this difference is that in task-switching paradigms with bivalent stimuli, participants must use the task specific pre-cues, else they would not know what task to perform and hence would have very high error rates. This is not true in congruency pre-cueing studies where pre-cues could be ignored completely and participants would still know what to do to avoid errors (e.g., in Stroop, name the color). Perhaps this suggests that a primary reason for lack of pre-cue benefits is that participants mostly ignore the cues. A second reason is that in task switching the contrast showing residual costs is typically between cues presented with varying CSIs, and not between an uncued and cued condition. To the extent that the uncued condition leads to similar preparation to the cued condition in congruency pre-cueing studies (a possibility considered in the section “Design Limitations”), there may not be a pre-cue benefit on incongruent trials. A third potential reason for the difference is that in task-switching, preparation is seemingly more concrete (e.g., activating task rules to press the left key if odd and right key if even when the parity task is cued) than that needed to produce a pre-cue benefit on incongruent trials in conflict tasks (abstract, distractor-attenuating strategy). Thus, it may be easier to observe some signature of preparation in task switching, even if it is not complete, than in the pre-cueing paradigm.

14 In a design with two tasks, a pre-cue signaling a switch essentially serves as a task specific pre-cue since a switch means the participant will perform the only other task that is not the one that was just performed (e.g., performed A → “switch” → must be followed by B). This is of course also true for pre-cues signaling a repeat in two-task and more than two-task designs.

15 Liu and Yeung (2020) employed a no-instruction condition in which lists were uncued and 50% switch, but the researchers did not compare these lists with the cued 50% switch lists. Given that their no-instruction (i.e., uncued) lists did not vary in switch probability, it is unclear if differences in comparison to the cued lists would be attributable to switch variability or pre-cues.

## Code availability

Not applicable (This is a theoretical/review article).
